# Achiral guest-mediated modulation of circularly polarized luminescence in chiral charge-transfer cocrystals

**DOI:** 10.1039/d6sc04089f

**Published:** 2026-07-13

**Authors:** Jialin Cui, Yu Wang, Yujie Liu, Hui Liu, Wei Wang, Yingjie Zhao

**Affiliations:** a State Key Laboratory of Advanced Optical Polymer and Manufacturing Technology, College of Polymer Science and Engineering, Qingdao University of Science and Technology 53 Zhengzhou Road Qingdao 266000 China hliu@qust.edu.cn yz@qust.edu.cn https://en.yz.qust.edu.cn/; b Shanghai Key Laboratory of Green Chemistry and Chemical Processes, State Key Laboratory of Petroleum Molecular and Process Engineering, School of Chemistry and Molecular Engineering, East China Normal University 3663 N. Zhongshan Road Shanghai 200062 China wwang@chem.ecnu.edu.cn

## Abstract

Chiral charge-transfer (CT) systems provide an important platform for the development of circularly polarized luminescence (CPL) materials. However, precise modulation of CPL performance remains challenging. Herein, we report a host–guest supramolecular assembly strategy based on a naphthalene diimide-derived triangular macrocycle to construct a series of chiral CT single-crystal systems. A variety of aromatic guest molecules co-assemble with the host macrocycle through π–π stacking interactions, forming highly ordered supramolecular architectures. Although the parent macrocycle exhibits pronounced circular dichroism (CD) signals, it is CPL-inactive. Remarkably, upon co-assembly with achiral guest molecules, strong CPL responses emerge, indicating efficient transfer of ground-state chirality into the excited state. Notably, the CPL intensity varies significantly among the single-crystal systems, with |*g*_lum_| values reaching up to 0.011. In addition, the emission color can be tuned from yellow to orange and red, enabling guest-dependent regulation of photophysical properties. Mechanistic studies reveal that multiple noncovalent interactions, particularly host–guest π–π stacking, cooperatively facilitate CT processes, thereby enabling effective modulation of excited-state chirality and CPL properties. This work provides a robust supramolecular strategy for constructing tunable chiral luminescent single-crystal materials.

## Abstract 2

Achiral guest molecules mediate the modulation of circularly polarized luminescence in chiral charge-transfer cocrystals through host–guest interactions, enabling tunable emission colors and enhanced CPL performance.

## Graphical abstract

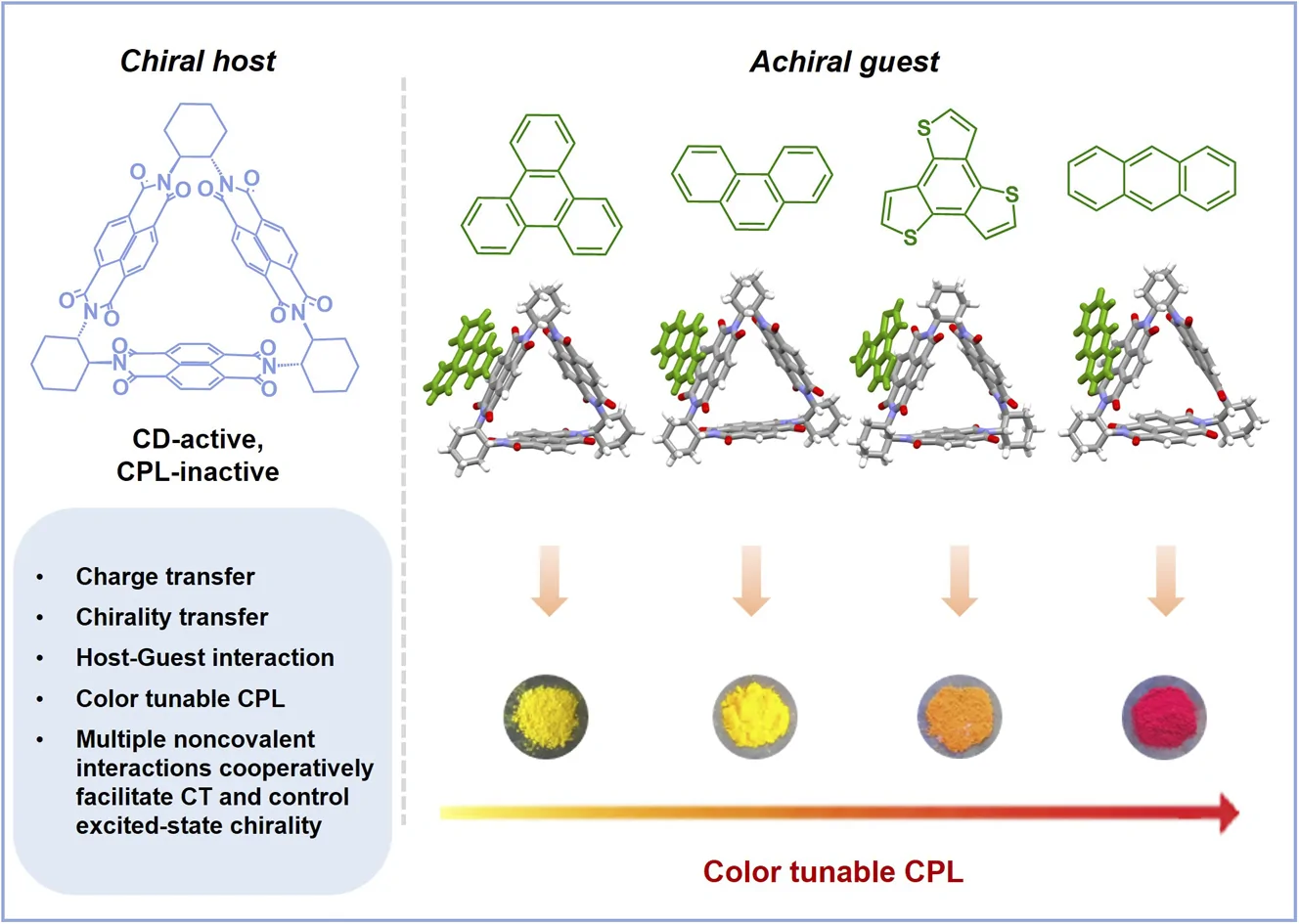

## Introduction

Chirality is a fundamental and ubiquitous property in nature, governing chiroptical phenomena and their hierarchical transfer across multiple length scales, and playing a pivotal role in systems ranging from biological assemblies to advanced functional materials.^1–5^ Against this backdrop, the study of chiroptical properties has become particularly important. Circularly polarized luminescence (CPL) has attracted increasing attention because of its broad potential in photonic devices, information encryption, bioimaging, and advanced optical displays.^6–9^ A central challenge in this field is how to efficiently translate molecular chirality into excited-state chiroptical emission and, more importantly, how to precisely tune CPL performance through rational structural design.^10–13^ Supramolecular assembly has emerged as an effective strategy for addressing this challenge.^14–20^ Compared with isolated chiral molecules, supramolecular systems can organize luminophores into ordered chiral architectures through noncovalent interactions, thereby amplifying chiral optical responses and enabling more efficient transfer of chirality from the molecular scale to higher-order assemblies.^21–23^ It is noteworthy that the well-defined binding modes in host–guest complexation enable controllable regulation of noncovalent interactions, thereby providing a higher level of structural precision.^24–26^ In host–guest assembly systems, weak intermolecular interactions, such as π–π stacking, hydrogen bonding, and donor–acceptor (D–A) interactions, play a decisive role in determining supramolecular packing, excited-state coupling, and nonradiative energy dissipation pathways, all of which are closely related to CPL performance.^27–29^ To date, most studies of luminescent materials constructed *via* host–guest complexation have primarily focused on fluorescence and phosphorescence, whereas studies on their CPL remain relatively rare.^30–32^ Therefore, the synergistic integration of molecular chirality with host–guest supramolecular organization provides a powerful strategy for the development of tunable CPL materials. Therefore, the synergistic integration of molecular chirality and supramolecular organization offers a powerful route toward tunable CPL materials.

Among the various intermolecular interactions used in supramolecular CPL systems, charge-transfer (CT) interactions between electron donors and electron acceptors are particularly attractive. On the one hand, D–A interactions can direct the formation of ordered co-assembled structures through intermolecular recognition and π-stacking. On the other hand, the resulting CT states can significantly reshape the excited-state electronic structure, thereby influencing absorption, emission, and chiroptical responses.^33–40^ Such dual structural and electronic functions make chiral CT assemblies highly promising platforms for CPL regulation. Nevertheless, the luminescence behavior of CT co-assembly systems is highly sensitive to subtle variations in the structure of guest molecules, including molecular size, π-planar geometry, and electron-donating ability.^41–44^ How these factors regulate supramolecular packing, donor–acceptor coupling, excited-state chirality, and ultimately CPL output remains insufficiently understood.

Herein, we report a series of chiral CT cocrystal systems constructed from a naphthalene diimide (NDI)-based triangular macrocycle (H1) and several aromatic donor guests through a host–guest supramolecular assembly strategy. By varying the guest molecules, distinct supramolecular packing modes, donor–acceptor interactions, and CT characteristics are achieved. While the parent macrocycle H1 shows clear circular dichroism (CD) signals but no detectable CPL activity, pronounced CPL responses emerge after co-assembly with the donor molecules, indicating that supramolecular CT assembly effectively promotes the transfer of chirality from the ground state to the emissive excited state. Moreover, the CPL intensity varies significantly among the cocrystal systems, revealing that guest-dependent supramolecular organization and donor–acceptor coupling play a crucial role in governing CPL behavior. This work demonstrates that the combination of molecular chirality, supramolecular assembly, and weak D–A interactions provides an effective strategy for regulating CPL properties and offers useful insight into the structure-packing-excited-state relationship in chiral CT cocrystal systems.

## Results and discussion

### Chiral charge-transfer cocrystal assembly

**Fig. 1:**
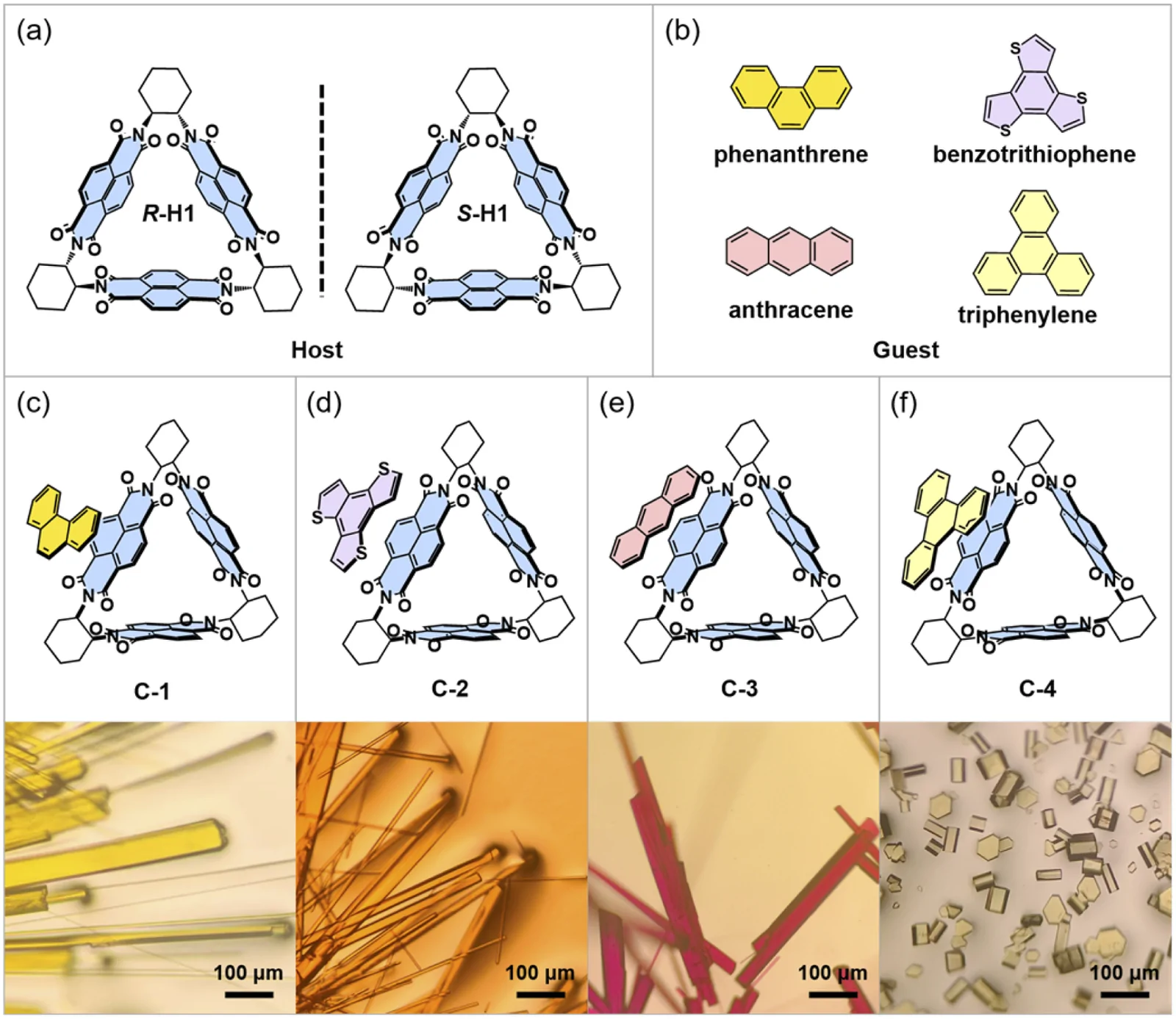
(a) Chemical structures of the enantiomeric electron-accepting host H1. (b) Chemical structures of the aromatic donor guests phenanthrene, benzotrithiophene, anthracene and triphenylene. (c–f) Chemical structures and corresponding optical microscopy images of the supramolecular assemblies C-1, C-2, C-3, and C-4. The structure of the *R* enantiomer is shown as a representative example.

The enantiomeric macrocyclic hosts *R*/*S*-H1 were synthesized through a one-pot condensation of naphthalene diimide with enantiomeric chiral cyclohexanediamine, whereby the molecular chirality of the diamine building blocks is embedded into the macrocyclic backbone (Fig. 1a).^45–47^ Meanwhile, as a typical electron-deficient building block, NDI endows the macrocyclic host *R*/*S*-H1 with three electron-deficient π-surfaces, thereby imparting to the macrocycle a distinctive structural feature that integrates chirality with electron deficiency.^48–50^ These characteristics facilitate the co-assembly of the macrocycle with suitably sized, electron-rich, planar π-donors *via* π–π stacking and D–A interactions, thereby yielding ordered supramolecular assemblies.^51–59^ Based on this structural design, the supramolecular chirality and chiroptical properties of the resulting assemblies can be modulated by varying the electron-donating ability of the guest molecules, which may in turn influence their CPL behavior.

To systematically investigate the co-assembly behavior of the chiral host H1, four aromatic guest molecules—phenanthrene (Phe), benzotrithiophene (BTT), anthracene (An) and triphenylene (Tpl)—were selected because of their distinct π-planar structures and electron-donating abilities (Fig. 1b). By carefully optimizing the crystallization solvent systems and diffusion conditions, a series of high-quality chiral CT cocrystals were successfully obtained, namely *R*/*S*-C-4, *R*/*S*-C-1, *R*/*S*-C-2, and *R*/*S*-C-3 (Fig. 1c–f). Detailed procedures for the self-assembly process are provided in the SI. During crystallization, the co-assembly systems exhibited pronounced color changes. The initially white or pale-colored host and guest components underwent a clear color transformation, giving rise to cocrystals with tunable colors spanning red, orange, yellow, and bright yellow (Fig. 1c–f and S3). These readily visible optical changes suggest the formation of strong CT interactions between the host and donor molecules, thereby providing a structural and electronic foundation for modulating their chiroptical properties.

**Fig. 2:**
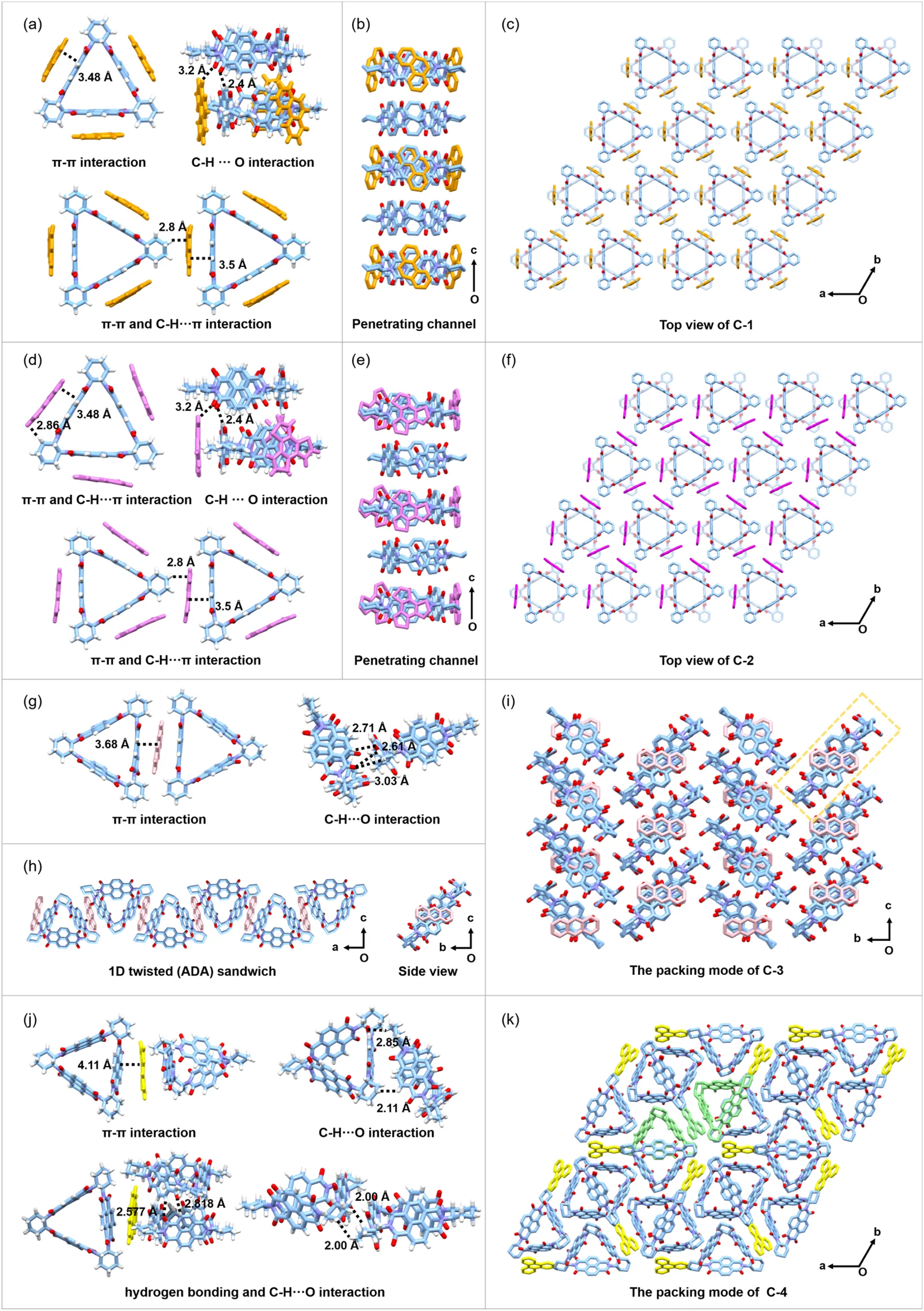
Single-crystal structures of C-1, C-2, C-3, and C-4. (a) π–π, C–H⋯π, and C–H⋯O noncovalent interactions in C-1. (b) Continuous supramolecular channels in C-1, viewed along the *a*-axis. (c) Packing mode of C-1 viewed along the *c* axis. (d) π–π, C–H⋯π, and C–H⋯O noncovalent interactions in C-2. (e) Continuous supramolecular channels in C-2, viewed along the *a*-axis. (f) Packing mode of C-2 viewed along the *c*-axis. (g) π–π and C–H⋯O noncovalent interactions in C-3. (h) 1D linear assembly of H1 and An formed through π–π interactions, and its side view. (i) Zigzag packing mode of C-3 viewed along the *c*-axis (the orange box highlights the side view of the 1D ADA chain formed by H1 and An). (j) π–π and C–H⋯O noncovalent interactions in C-4. (k) Packing mode of C-4 viewed along the *c*-axis (the molecule highlighted in green represents one ADA building unit). The crystal structure is described using the *R* enantiomer as a representative example. The macrocyclic host is shown in blue, with oxygen, nitrogen, and hydrogen atoms represented in red, purple, and white, respectively. To distinguish different electron-rich guest molecules, Phe, BTT, An, and Tpl are colored orange, purple, pink, and yellow, respectively. Hydrogen bonds and other intermolecular interactions are depicted as black dashed lines. For clarity, hydrogen atoms are omitted in the packing diagrams and selected repeating stacking motifs, and solvent molecules in the crystal structures are also omitted.

All single-crystal structures were solved by single-crystal X-ray diffraction (SCXRD) and belong to chiral space groups (Table S1). The results reveal that subtle variations in the guest molecules can induce significant changes in the assembly modes of the resulting host–guest supramolecular crystals, leading to distinctly different supramolecular packing arrangements (Fig. 2). The C-1 cocrystal adopts a layered supramolecular assembly mode featuring channel-like structures. Within a single layer, each H1 molecule associates with three Phe molecules through its three NDI π-faces *via* π–π stacking interactions (3.48–3.50 Å), thereby forming a stable two-dimensional (2D) supramolecular layer (Fig. 2a). Adjacent to this layer is another 2D layer composed exclusively of H molecules. These neighboring layers are interconnected through multiple C–H⋯O interactions, including both H1⋯H1 and H1⋯Phe contacts (2.4–3.2 Å), and propagate periodically along the *c*-axis (Fig. 2b). This assembly mode generates channel-like structures along the *c-*axis, with the channels being defined by the intrinsic through-cavity of H1. Furthermore, adjacent channels are additionally stabilized by π–π stacking between H1 and Phe molecules (3.48–3.50 Å), together with C–H⋯π interactions (2.8 Å), ultimately resulting in a porous supramolecular architecture with an AB stacking mode (Fig. 2c). The supramolecular assembly mode of C-2 is highly analogous to that of C-1 (Fig. 2d–f). In the C-2 cocrystal, π–π stacking, C–H⋯π, and C–H⋯O interactions between H1 and BTT molecules cooperatively construct continuous channels, thereby further stabilizing the supramolecular architecture.

As for the other two supramolecular assemblies, C-3 and C-4 adopt distinct assembly modes. In the C-3 cocrystal, only two of the three NDI π-faces of each H1 molecule participate in π–π interactions with An molecules (3.68 Å), giving rise to a one-dimensional (1D) linear supramolecular assembly arrangement (Fig. 2g and h). These linear chains are further interconnected through C–H⋯O interactions between neighboring H1 molecules (2.71–3.03 Å), resulting in a zigzag packing mode (Fig. 2i). In the C-4 cocrystal, only one NDI π-face of H1 participates in π–π interactions with the electron-donating Tpl molecules, thereby forming the basic host–guest assembly units (Fig. 2j). The H1 molecules are further linked through C–H⋯O interactions (2.85 Å) and hydrogen bonds (2.00–2.11 Å). In addition, C–H⋯O interactions (2.36 Å) between H1 and adjacent Tpl molecules further stabilize the overall packing, giving rise to the structure shown in Fig. 2k.

To further corroborate the structural reliability of the cocrystals, powder X-ray diffraction (PXRD) and thermogravimetric analysis (TGA) were conducted (Fig. S4 and S5). The PXRD patterns of all cocrystals are in good agreement with the simulated profiles, confirming their high crystallinity and phase purity. Meanwhile, the TGA results show that all samples exhibit negligible weight loss below 100 °C, indicating the absence of residual solvents or adsorbed moisture. A gradual mass loss is observed in the range of 100–350 °C, which can be attributed to the removal of weakly bound components, followed by a pronounced weight loss between 400 and 500 °C corresponding to the decomposition of the main structure. Collectively, these results demonstrate that the cocrystals possess excellent thermal stability.

### Charge-transfer-induced photophysical properties of the cocrystals

**Fig. 3:**
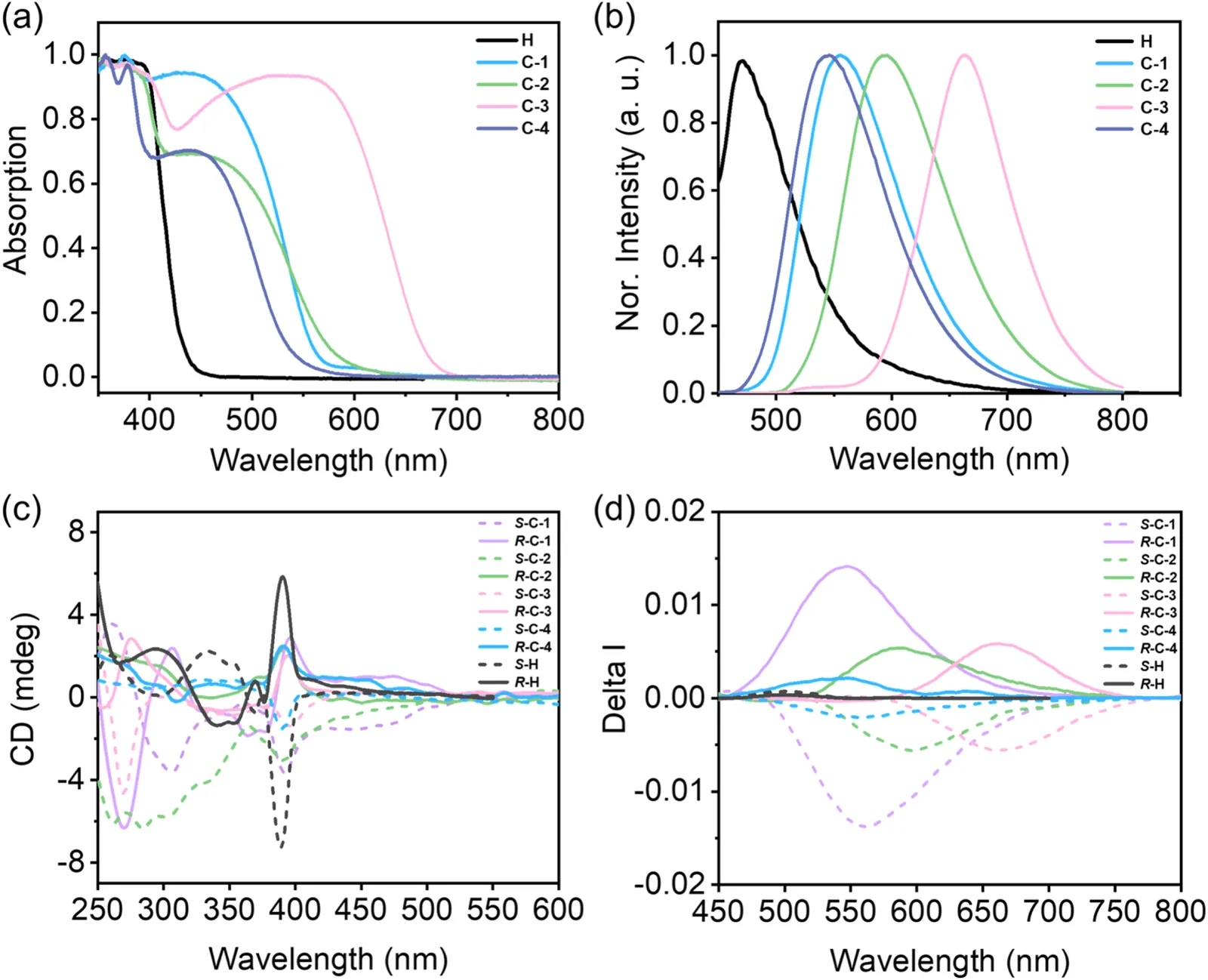
(a) Normalized UV/vis absorption spectra of H1 and the cocrystals C-1, C-2, C-3, and C-4. (b) Normalized PL spectra of H1 and the corresponding cocrystals. (c) CD spectra of *R*/*S*-H1 and *R*/*S*-C-1, *R*/*S*-C-2, *R*/*S*-C-3, and *R*/*S*-C-4. (d) CPL spectra of *R*/*S*-H1 and the corresponding cocrystals. UV/vis absorption spectra and PL spectra were measured using the *R* enantiomeric single crystals as representative samples.

The photophysical properties of the cocrystals, which exhibit distinctly different colors visible to the naked eye, were subsequently investigated. To clarify the origin of these color variations, the solid-state UV-vis absorption spectra of the four cocrystals and their constituent host and guest molecules were measured (Fig. 3a and S6). Compared with the H1 host (400 nm), all cocrystals display red-shifted absorption edges, located at 675 nm, 585 nm, 560 nm, and 540 nm for C-3, C-2, C-1, and C-4, respectively (Fig. 3a). The optical band gaps of the four cocrystals were further estimated from the Tauc plots (Fig. S7). The corresponding band gap values are 2.55 eV, 2.70 eV, 2.74 eV, and 3.02 eV for C-3, C-2, C-1, and C-4, respectively. Notably, the trend in band gaps is consistent with both the variation in the absorption edges observed in the UV-vis spectra and the distinct color changes visible to the naked eye, further confirming the close correlation between optical band gap modulation, electronic structure variation, and the host–guest interactions in these cocrystals.

Consistent with the absorption results, the emission spectra also exhibit a donor-dependent variation (Fig. 3b). C-3, C-2, C-1, and C-4 display single emission peaks at 662 nm, 594 nm, 556 nm, and 544 nm, respectively, and all cocrystal systems show a pronounced red shift relative to H1 (470 nm). Time-resolved photoluminescence measurements were further conducted to investigate the excited-state dynamics (Fig. S8). The fluorescence lifetimes of the cocrystals are 9.64 ns for C-1, 7.09 ns for C-2, 7.97 ns for C-3, and 12.60 ns for C-4. These differences in fluorescence lifetimes indicate that the nature of the guest molecules has a significant influence on the excited-state dynamics of the cocrystals. The absolute photoluminescence quantum yields of C-1, C-2, C-3, and C-4 were measured to be 24.3%, 21.4%, 20.0%, and 20.8%, respectively. Overall, these results indicate that the four cocrystals exhibit pronounced CT characteristics.^60^ The distinct electron-donating abilities and molecular sizes of the guest molecules significantly affect intermolecular packing and donor–acceptor arrangement, thereby modulating the CT interaction strength, energy-level structures, and photophysical properties of the cocrystals. Such donor–acceptor co-assembly also provides a suitable platform for investigating their guest-dependent CPL behavior.

Subsequently, the chiroptical properties of these cocrystals were systematically investigated. Thin films of the chiral cocrystals were fabricated by drop-casting concentrated solutions of the corresponding compounds onto quartz substrates, and their ground-state chirality was probed by CD spectroscopy. As shown by the black traces in Fig. 3c, the chiral macrocycle *R*/*S*-H1 exhibits well-defined and persistent ground-state chirality, displaying pronounced Cotton effects at 333, 359, 368, and 389 nm. These CD signals are in excellent agreement with the features observed in the solid-state UV/vis absorption spectrum of H1, indicating that the chirality arises from the conformational asymmetry induced by the cyclohexanediamine units and is efficiently transmitted across the π-conjugated system (Fig. S11). While the co-assembled systems *R*/*S*-C-3, *R*/*S*-C-2, *R*/*S*-C-1, and *R*/*S*-C-4 exhibit multiple sets of mirror-symmetric CD signals in the 380–480 nm region, which are markedly red-shifted relative to those of the parent triangular macrocycle. This bathochromic shift is attributed to the formation of CT interactions between the host and guest, which modulate the electronic distribution and reorganize the excited-state energy levels. These observations indicate that, upon supramolecular co-assembly, the molecular chirality encoded in the cyclohexanediamine units of H1 is effectively transferred to the CT cocrystals and further amplified through cooperative π–π stacking and electrostatic interactions (Fig. 3c).^61^ In addition, all four CT cocrystals exhibit pronounced CPL signals, and the *R* and *S* enantiomers of each cocrystal show well-defined mirror-image relationships in their CPL responses (Fig. 3d). The excitation wavelength (*λ*_ex_) was 405 nm for all fluorescence and CPL measurements. Specifically, the |*g*_lum_| values for *R*/*S*-C-3, *R*/*S*-C-2, *R*/*S*-C-1, and *R*/*S*-C-4 are 0.006, 0.005, 0.011, and 0.005, respectively (Fig. S12). This observation indicates that the system retains a highly symmetric chiroptical response in the excited state, demonstrating that chirality is not only effectively transferred in the ground state but also preserved and expressed during excited-state dynamics.

Furthermore, we investigated the CT interactions between the host and guest in solution. The UV-vis absorption spectrum recorded in dichloromethane shows no evident red-shifted absorption bands or new CT features compared with the individual components (Fig. S13). We further examined the system in acetonitrile to evaluate the effect of solvent polarity on CT interactions; however, no clear CT signatures were observed in either case (Fig. S14). These results suggest that, in solution phase, the host–guest interactions are relatively weak and insufficient to promote the formation of stable, ordered supramolecular assemblies. Consistently, no discernible CPL signals were detected in solution at concentrations of 10^−5^ M and 10^−3^ M (Fig. S15 and S16). Overall, these observations indicate that the pronounced CT characteristics and CPL responses observed in the solid state originate from the cooperative enhancement of multiple noncovalent interactions within the crystal packing.

Although all cocrystals display discernible CPL signals, the host triangular macrocycle H1 exhibits only pronounced CD signals and weak fluorescence emission, with no measurable CPL activity (black traces in Fig. 3c and d). This comparison highlights that the host–guest complexation strategy enables precise modulation of the CT process and the evolution of excited-state chirality, thereby providing an effective approach to fine-tune the CPL performance of chiral luminescent systems. This marked contrast originates from the fundamental difference in the nature of the excited states. In the isolated H1 macrocycle, the emission is predominantly governed by localized excited states, where limited electronic redistribution results in inefficient coupling between electric and magnetic transition dipole moments. Consequently, despite its well-defined ground-state chirality, H1 fails to generate observable CPL. In contrast, upon co-assembly with guest molecules, CT excited states are formed, featuring enhanced electronic delocalization and increased sensitivity to the chiral environment. This facilitates more effective coupling between transition dipole moments, thereby enabling CPL emission. Cocrystals assembled with different guest molecules exhibit pronounced variations in CPL behavior, which can be attributed to the synergistic interplay among guest-dependent supramolecular packing, CT coupling, and excited-state energy dissipation. Distinct guest molecules induce markedly different host–guest assembly modes, leading to variations in donor–acceptor arrangements and chiral organization. In particular, the number of NDI π-faces involved in π–π interactions plays a critical role in determining the extent of electronic coupling and the efficiency of chirality transfer to the excited state. Meanwhile, the size and electronic properties of the guest molecules govern their geometric compatibility with the triangular macrocycle H1, thereby modulating both packing order and CT interaction strength. Overall, this comparison demonstrates that the host–guest complexation strategy enables precise modulation of excited-state chirality and CT processes through tailored supramolecular organization, offering an effective approach for fine-tuning the CPL performance of chiral luminescent systems.

### Theoretical calculations

**Fig. 4:**
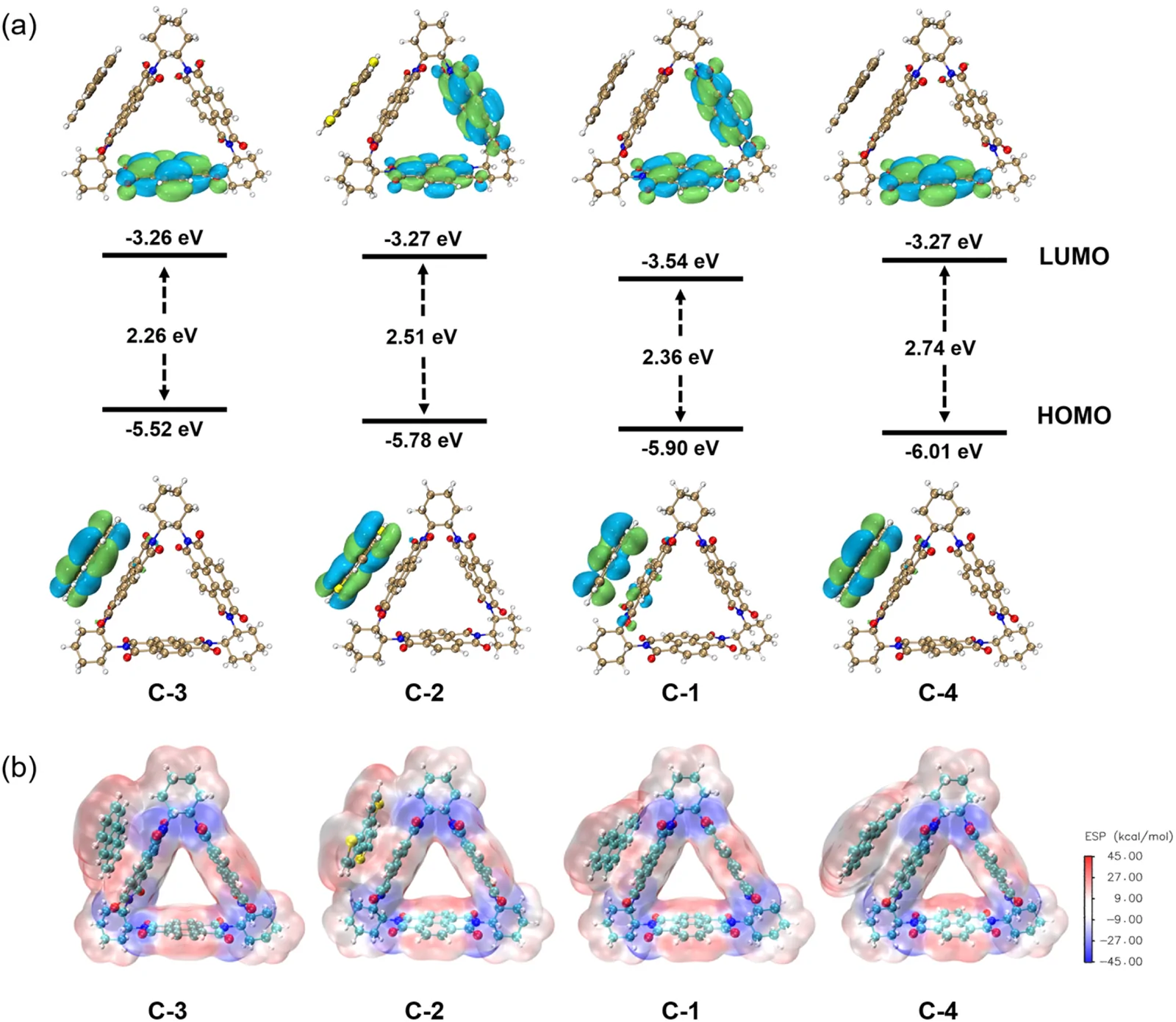
(a) HOMO, LUMO, and HOMO–LUMO energy gaps of the D–A fragments in the C-3, C-2, C-1, and C-4 cocrystal systems. (b) ESP surfaces mapped on the van der Waals surfaces of C-3, C-2, C-1, and C-4 (range: −45 to +45 a.u.). Red and blue regions denote electron-rich and electron-deficient areas, respectively. A consistent potential scale is used for comparison. Calculations were carried out using the *R* enantiomeric single crystal structure as a model.

To obtain a more detailed understanding of how the guest molecules regulate CPL, quantum chemical calculations were performed based on the single-crystal structures of the CT cocrystals (Fig. 4). The corresponding highest occupied molecular orbital (HOMO)-lowest unoccupied molecular orbital (LUMO) energy levels and frontier orbital distributions were obtained from geometries optimized from the crystallographic structures (Fig. 4a). The HOMO energy levels of the four cocrystals, *R*/*S*-C-3, *R*/*S*-C-2, *R*/*S*-C-1, and *R*/*S*-C-4, are predominantly localized on the respective guest molecules (An, BTT, Phe, and Tpl), with values of −6.01 eV, −5.09 eV, −5.78 eV, and −5.52 eV, respectively, showing a gradual variation across the series (Fig. 4a and S17). In contrast, the LUMO levels of these systems are relatively similar and mainly distributed on the host molecule H1, with an energy of approximately −2.58 eV, consistent with a CT process from the guest to the H1 acceptor unit. Notably, as the HOMO levels of the guest molecules vary, the HOMO–LUMO energy gaps can be tuned from 2.26 to 2.74 eV, indicating that the CT strength plays a crucial role in modulating the photophysical properties of the host–guest complexes. Accordingly, the CPL emission of the present system can be effectively regulated by selecting guests with different electron-donating abilities. Electrostatic potential (ESP) analysis further reveals the nature of host–guest interactions (Fig. 4b). The ESP surfaces show complementary distributions between electron-rich regions of the guest molecules and electron-deficient regions of the H1 host, providing favorable electrostatic driving forces for host–guest recognition and charge-transfer processes. Variations in π-conjugation extent, electron density, and electron-donating ability among the guest molecules lead to distinct ESP distributions, thereby modulating the strength of intermolecular interactions and CT efficiency. The spatial separation of HOMO and LUMO indicates a CT character, modulated by the electronic properties of the guest molecules. In addition, the spatial distribution of the LUMO over the triangular macrocycle is not identical among the different cocrystals, which can be attributed to the modulation of the acceptor π-electron system by the guest molecules. Differences in π-conjugation extent, electron density, and electron-donating ability of the guests influence the electronic coupling strength between host and guest units, resulting in varying degrees of orbital overlap.

## Conclusion

In summary, we have constructed a series of chiral CT cocrystals through a host–guest co-assembly strategy based on a triangular NDI macrocycle H1 and various achiral aromatic guests. By modulating the size and electron-donating ability of the guest molecules, distinct supramolecular packing modes and donor–acceptor arrangements were achieved, leading to tunable photophysical and chiroptical properties. Notably, while the parent macrocycle H1 exhibits only CD activity without detectable CPL, the formation of CT cocrystals gives rise to pronounced CPL emission, highlighting the crucial role of guest-induced CT excited states in activating and amplifying chiral luminescence. Systematic investigations reveal that the CPL performance is governed by a delicate balance among supramolecular packing, CT coupling, and excited-state energy dissipation. In particular, the extent of π–π interactions, together with host–guest packing modes and CT coupling, collectively determines the efficiency of chirality transfer to the excited state and the resulting emission behavior. Overall, this work not only provides important insights into the structure–property relationships in CT-based chiral luminescent systems, but also demonstrates that host–guest complexation represents an effective strategy for modulating excited-state chirality and optimizing CPL performance, thereby offering new guidelines for the rational design of advanced chiroptical materials.

## Author contributions

Y. Zhao conceived the idea and designed the experiments. J. Cui performed the experiments, carried out the synthesis and single-crystal growth, and conducted the single-crystal X-ray analysis and theoretical calculations. Y. Wang performed all CPL measurements. H. Liu supervised the synthesis and single-crystal growth. W. Wang supervised the CPL studies. Y. Zhao supervised the project.

## Conflicts of interest

There are no conflicts to declare.

## Supplementary Material

SC-017-D6SC04089F-s001

SC-017-D6SC04089F-s002

## Data Availability

CCDC 2544382–2544389 contain the supplementary crystallographic data for this paper.^62*a*–*h*^ Supplementary information: Experimental procedures and spectroscopic data, including Table S1 and Fig. S1–S17. See DOI: https://doi.org/10.1039/d6sc04089f.
