# Supplementary material for: Achiral guest-mediated modulation of circularly polarized luminescence in chiral charge-transfer cocrystals
Source: Chem Sci. 2026 Jul 13;17(34):16506–14. doi: 10.1039/d6sc04089f (PMC13384016; doi:10.1039/d6sc04089f)
Supplement: Supplementary file 1 [file SC-017-D6SC04089F-s001.pdf]

Supplementary Information (SI) for Chemical Science.
This journal is © The Royal Society of Chemistry 2026

Supplementary Information

Achiral guest-mediated modulation of circularly polarized luminescence in chiral charge-transfer cocrystals

Jialin Cui,[a] Yu Wang,[b] Yujie Liu,[a] Hui Liu,*[a] Wei Wang,*[b] and Yingjie Zhao*[a]

[a] State Key Laboratory of Advanced Optical Polymer and Manufacturing Technology, College of Polymer Science and Engineering, Qingdao University of Science and Technology, 53 Zhengzhou Road, 266000, Qingdao, China

[b] Shanghai Key Laboratory of Green Chemistry and Chemical Processes, School of Chemistry and Molecular Engineering, East China Normal University, 3663 N. Zhongshan Road, Shanghai 200062, China.

[*] Corresponding Authors' E-mail: hliu@qust.edu.cn; wwang@chem.ecnu.edu.cn; yz@qust.edu.cn

Contents

1. Supporting Methods S2
1.1 General materials and methods S2
1.2 Synthesis procedure S3
1.3 Crystal growth and characterization S3
1.4 The single-crystal data of cocrystal S5
1.5 Preparation of chiral thin films S7
1.6 Computational Details S7
2. Supporting Figures S8
3. Reference S11

1. Supporting Methods

1.1 General materials and methods

Unless otherwise indicated, all other reagents are commercially purchased and used without further purification. Organic solvents including methanol, chloroform, dichloromethane (CH_2Cl_2), N,N-dimethylformamide (DMF), and hexane were purchased from Tansoole. Naphthalene tetramethylanhydride, *(RR)*- and *(SS)-trans*-1,2-cyclohexanediamine, anthracene, benzotrithiophene, phenanthrene, and triphenylene were purchased from Energy Chemical.

^{1}H and ^{13}C NMR spectra were recorded at 400 MHz and 101 MHz, respectively, on a Bruker AVANCE NEO 400 Ascend spectrometer in the indicated solvents at room temperature. Chemical shifts were reported in δ (ppm) relative to TMS (δ = 0).

The solid-state UV-Vis absorbance was measured by UV spectrophotometer (Agilent, cary5000).

The transmitted UV-Vis absorbance was measured by a UV spectrometer (HITACHI, 3900).

Circular dichroism (CD) and Circularly Polarized Luminescence (CPL) spectra were measured on a Chirascan Series Spectrometer (Applied Photophysics Ltd, UK) at room temperature. Both the solution and solid-state CD tests were operated in transmittance mode.

Single-crystal data were measured using a Bruker D8 VENTURE X-ray single-crystal diffractometer and a Rigaku XtaLAB Synergy. The refinement and analysis were carried out using Olex2-1.3 software. The molecular stacking patterns were calculated using Mercury software (copyright by CCDC).

The fluorescence quantum yield was measured using the Quantaurus-QY (Hamamatsu Photonics) instrumen.

The crystal morphology was observed using a microscope (Nikon ECLIPSE LV100ND POL, Japan).

1.2 Synthesis procedure

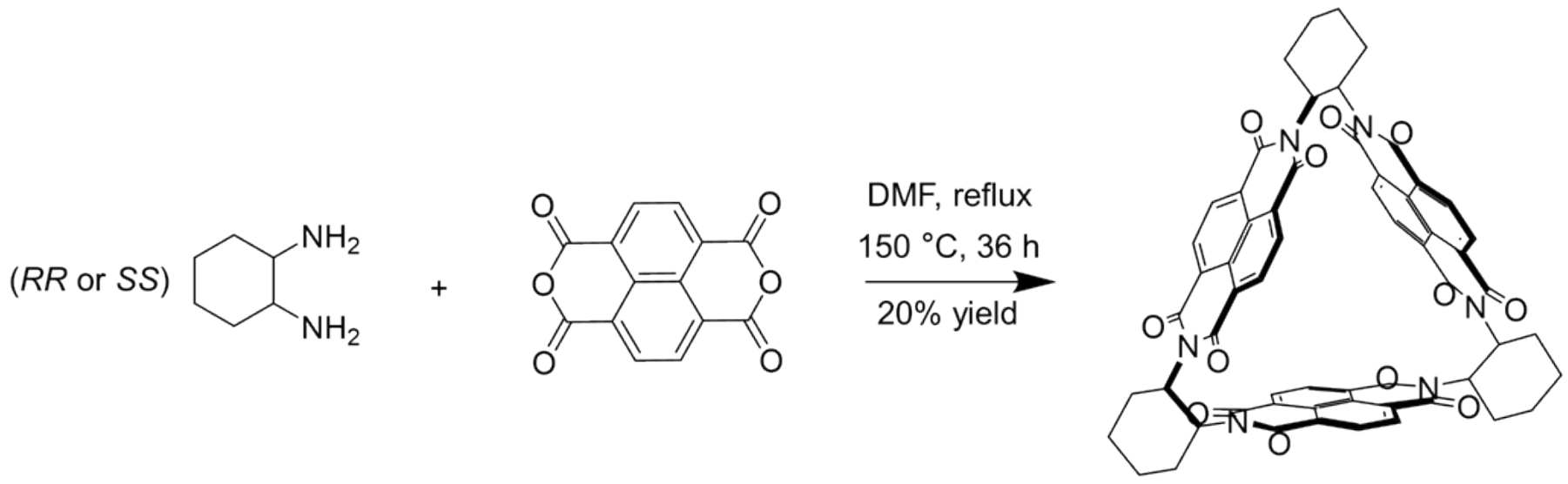

Scheme 1. Synthesis of compound *R* or *S*-H1.[1]

R or *S*-H1. Naphthalene tetracarboxylic dianhydride (12.3 g, 45.8 mmol) was dispersed in 450 mL of DMF in a 1 L single-neck flask equipped with a reflux condenser. At room temperature, (*RR or SS*)-trans-1,2-cyclohexanediamine (5.2 g, 45.8 mmol) was added to the suspension. The reaction mixture was then heated to reflux at 150 °C and maintained for 36 h. After completion of the reaction, DMF was removed under reduced pressure. The resulting red solid was soaked in a dichloromethane/methanol (100:1, v/v) mixture and sonicated for 10 min. The mixture was subsequently filtered, and the filtrate was concentrated under reduced pressure. The crude product was further purified by column chromatography using dichloromethane as the eluent (200:1, v/v). The collected eluate was concentrated to approximately half of its original volume, followed by the addition of ten volumes of methanol. The resulting mixture was allowed to stand overnight, leading to the formation of a white precipitate. The precipitate was collected and identified as *R* or *S*-H1 (3.2 g, 3.1 mmol) with a yield of 20%. This compound has been previously reported. ^{1}H NMR (400 MHz, $CDCl_3$) 8.49 (d, *J* = 1.5 Hz, 12H), 6.26 – 6.19 (m, 6H), 2.49 (d, *J* = 11.2 Hz, 6H), 2.03 – 1.91 (m, 14H), 1.75 – 1.61 (m, 6H). ^{13}C NMR (100 MHz, $CDCl_3$): 162.78, 162.05, 131.38, 130.78, 126.48, 126.12, 125.86, 53.93, 29.96, 25.75.

1.3 Crystal growth and characterization

R/S-H-Phenanthrene (*R/S*-C-1)

H1 (12.5 mg, 0.012 mmol) and phenanthrene (6.4 mg, 0.036 mmol) were dissolved in $CHCl_3$ (4 mL) to give a clear solution. n-Hexane was slowly diffused into the solution. After several days, high-quality yellow rod-like single crystals were obtained.

R/S-H-Benzotrithiophene (*R/S*-C-2)

H1 (12.5 mg, 0.012 mmol) and benzotrithiophene (8.9 mg, 0.036 mmol) were dissolved in $CHCl_3$ (4 mL)

to afford a clear solution. n-Hexane was slowly diffused into the solution. After several days, high-quality orange rod-like single crystals were obtained.

***R/S*-H-Anthracene (*R/S*-C-3)**

H1 (12.5 mg, 0.012 mmol) and anthracene (21.4 mg, 0.120 mmol) were dissolved in THF (4 mL) to afford a clear solution. Diethyl ether was then slowly diffused into the solution. After standing undisturbed for several days, high-quality red rod-like single crystals were obtained.

***R/S*-H-Triphenylene (*R/S*-C-4)**

H1 (12.5 mg, 0.012 mmol) and triphenylene (8.2 mg, 0.036 mmol) were dissolved in THF (4 mL). The solution was allowed to evaporate slowly at room temperature. After several days, high-quality rod-like single crystals were obtained.

1.4 The single-crystal data of cocrystals

Table S1. Crystal data and structure refinement.

CCDC	2544383	2544385	2544384	2544386
Identification code	**C-1** ***(S)***	**C-2** ***(S)***	**C-3** ***(S)***	**C-4** ***(S)***
Empirical formula	$C_{168}H_{120}Cl_{18}N_{12}O_{24}$	$C_{64.5}H_{58.5}Cl_{10.5}N_4O_8S_3$	$C_{81}H_{67}C_{l3}N_6O_{12}$	$C_{83}H_{77.8}N_6O_{16.4}$
Temperature/K	100	100	293	100
Space group	*P*321	*P*3	$P2_12_12_1$	*P*321
a/Å	19.1751	19.1861	10.3993	27.5399
b/Å	19.1751	19.1861	23.5965	27.5399
c/Å	15.5051	15.4486	27.0770	16.4027
α/°	90	90	90	90
β/°	90	90	90	90
γ/°	120	120	90	120
Volume/Å^3	4937.2	4924.90	6644.35	10773.8
Z	1	3	4	6
Data completeness	1.000	0.970	1.000	1.000
R(reflections)	0.0918	0.1086	0.0484	0.1232
wR2(reflections)	0.2771	0.3044	0.1416	0.3572

CCDC	2544382	2544389	2544388	2544387
Identification code	**C-1** ***(R)***	**C-2** ***(R)***	**C-3** ***(R)***	**C-4** ***(R)***
Empirical formula	$C_{168}H_{120}Cl_{18}N_{12}O_{24}$	$C_{161.5}H_{123.5}Cl_{30}N_{12}O_{24}S_6$	$C_{81}H_{67}C_{l3}N_6O_{12}$	$C_{85}H_{80}N_6O_{17}$
Temperature/K	100	100	293	100
Space group	*P*321	*P*1	$P2_12_12_1$	*P*321
a/Å	19.1753	15.6078	10.4175	27.5686
b/Å	19.1753	17.6829	23.5815	27.5686
c/Å	15.4885	18.9658	27.0506	16.3914
α/°	90	113	90	90
β/°	90	93	90	90
γ/°	120	116	90	120
Volume/Å^3	4932.0	4187.4	6645.26	10788.9
Z	1	1	4	6
Data completeness	0.990	0.990	1.000	0.990
R(reflections)	0.1192	0.0916	0.0562	0.1158
wR2(reflections)	0.3485	0.0972	0.1546	0.3451

1.5 Preparation of chiral thin films

R or *S*-H1 powder (2.0 mg, 1.936 μmol) was dissolved in dichloromethane (1.0 mL). The solution was filtered through a 0.45 μm microporous membrane to remove trace insoluble impurities, affording a clear solution with a concentration of 1.9 mM. Chiral thin films of *R/S*-H1 were then prepared by drop-casting the dichloromethane solution onto a quartz substrate and allowing the solvent to evaporate naturally.

Thin films used for CD and CPL measurements, as well as UV–vis absorption spectroscopy, were prepared using the same procedure. In these cases, solutions with concentrations of approximately 2.0 mM were drop-cast onto quartz substrates to form the corresponding films.

1.6 Computational Details

The energy levels were calculated using density functional theory (DFT) with the Gaussian 16 software package.[2] The structures of the donor–acceptor (D–A) molecular pairs were directly extracted from the crystal structure files, and the molecular orbitals were calculated at the B3LYP/6-31G(d,p) level of theory. The frontier molecular orbital isosurfaces were visualized using Multiwfn and VMD.[3]

2. Supporting Figures

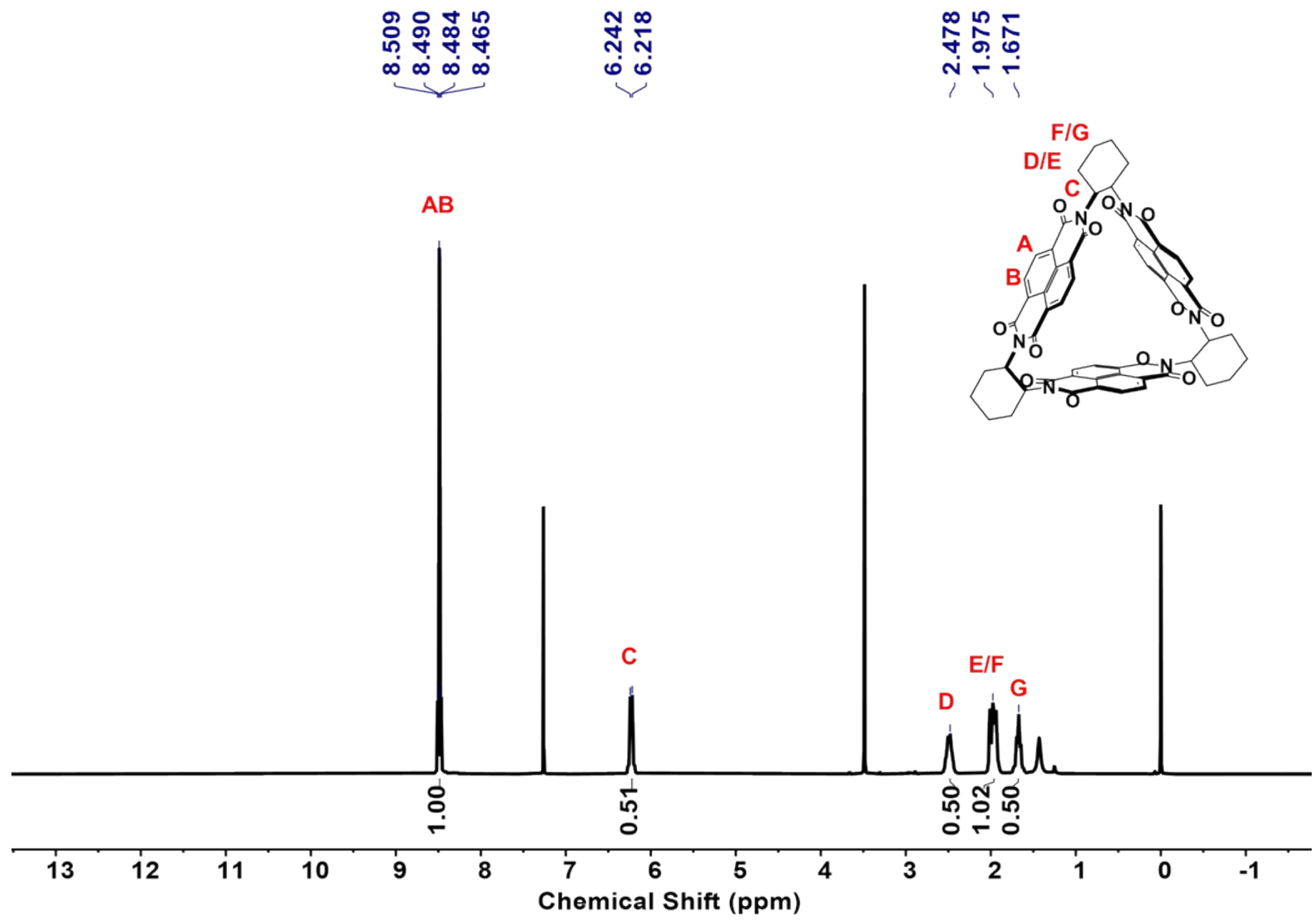

Figure S1. ^{1}H NMR spectrum (400 MHz, 298 K, $CDCl_3$) of *R/S*-H1.

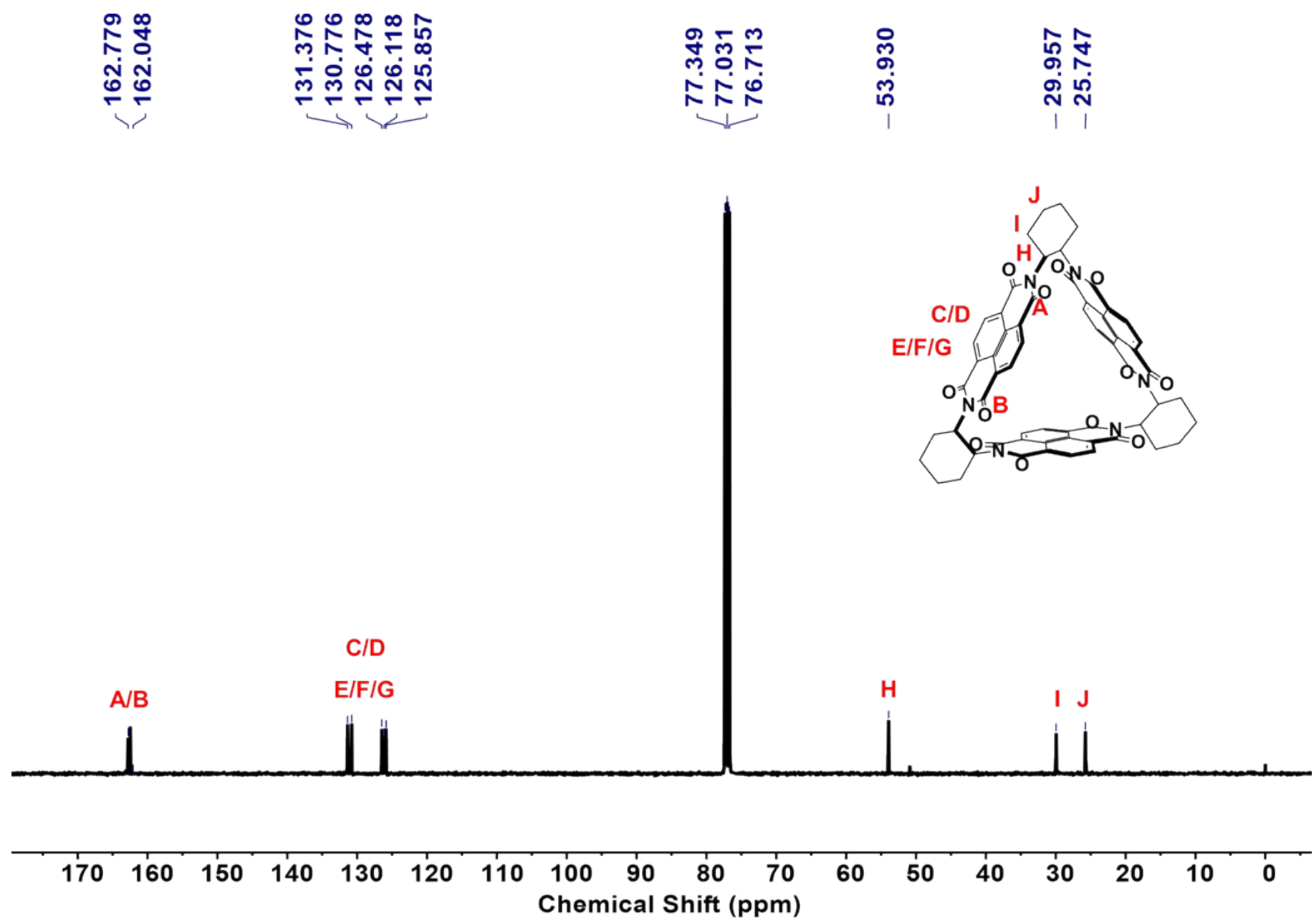

Figure S2. ^{13}C NMR spectrum (101 MHz, 298 K, $CDCl_3$) of *R/S*-H1.

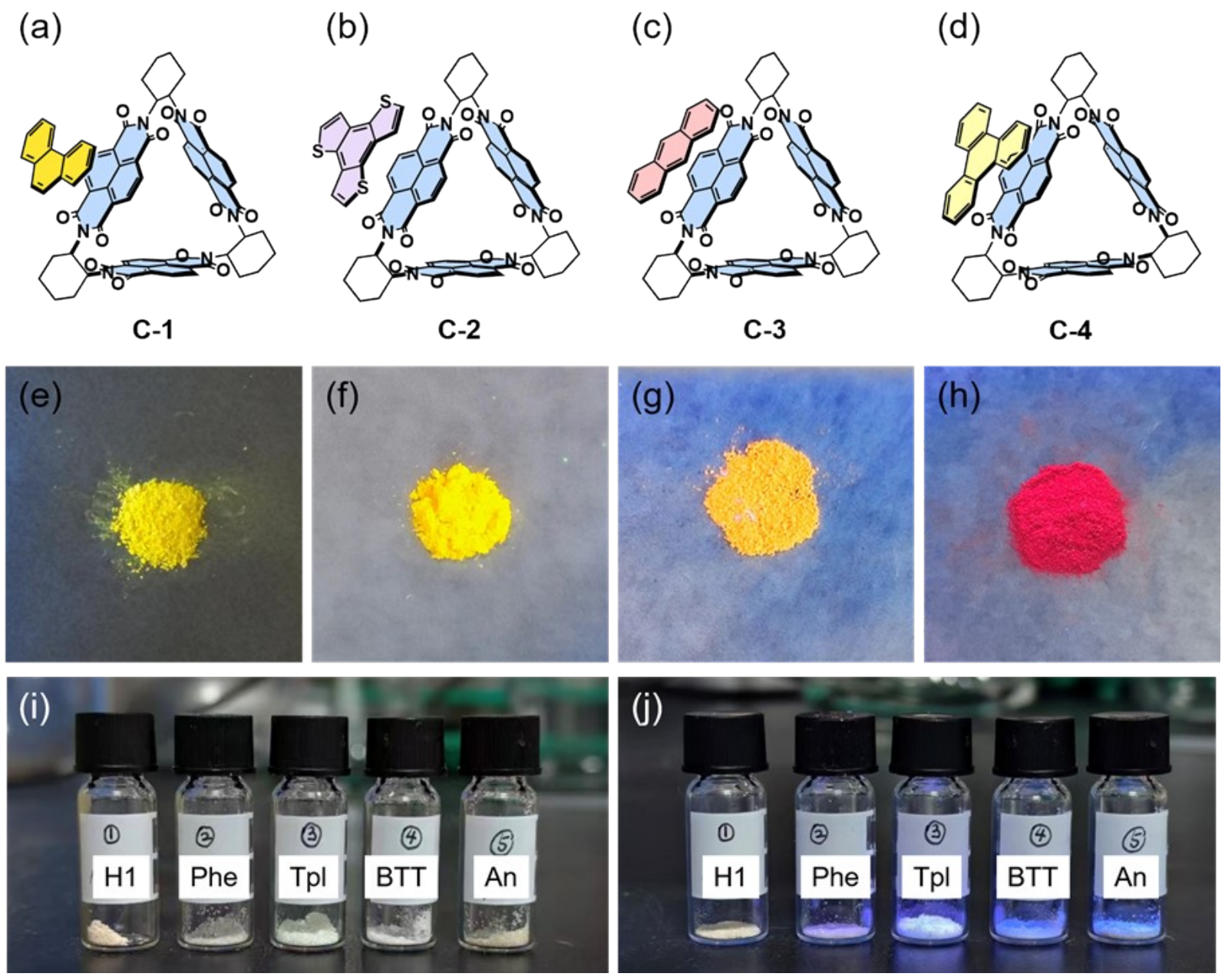

Figure S3. Structural and optical images of supramolecular assemblies. (a-d) Chemical structures of the supramolecular assemblies C-1, C-2, C-3, C-4. (e-h) Photographs of the assemblies under 365 nm UV light irradiation. (i) Daylight photographs of the electron acceptor H1 together with the electron donors Phe, Tpl, BTT, and An (from left to right). (j) Photographs of the electron donors under 365 nm UV light irradiation. Taking the *R* enantiomer as an example.

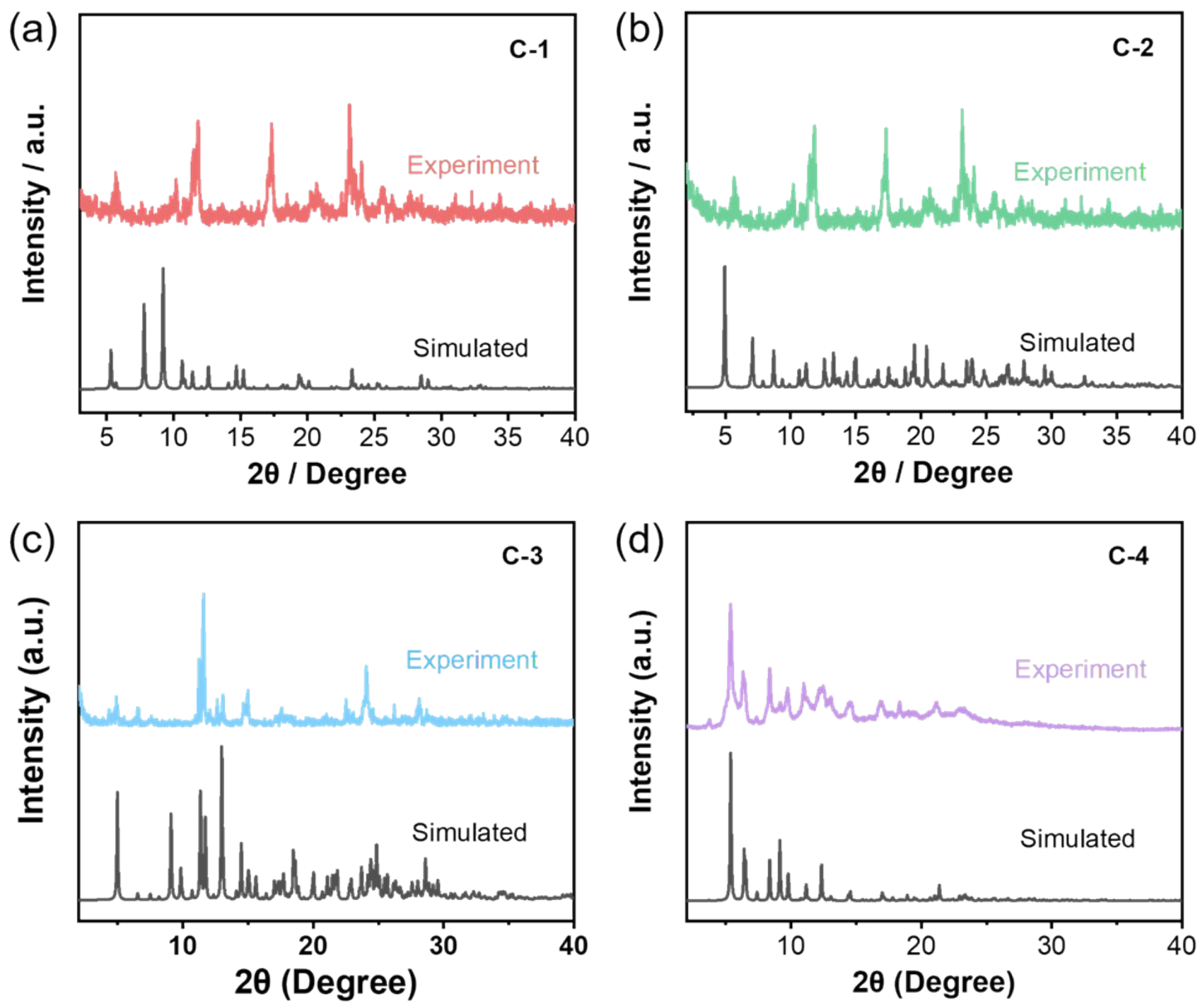

Figure S4. Powder X-ray Diffraction (PXRD) spectra of (a) C-1, (b) C-2, (c) C-3, and (d) C-4. Taking the *R* enantiomer as an example.

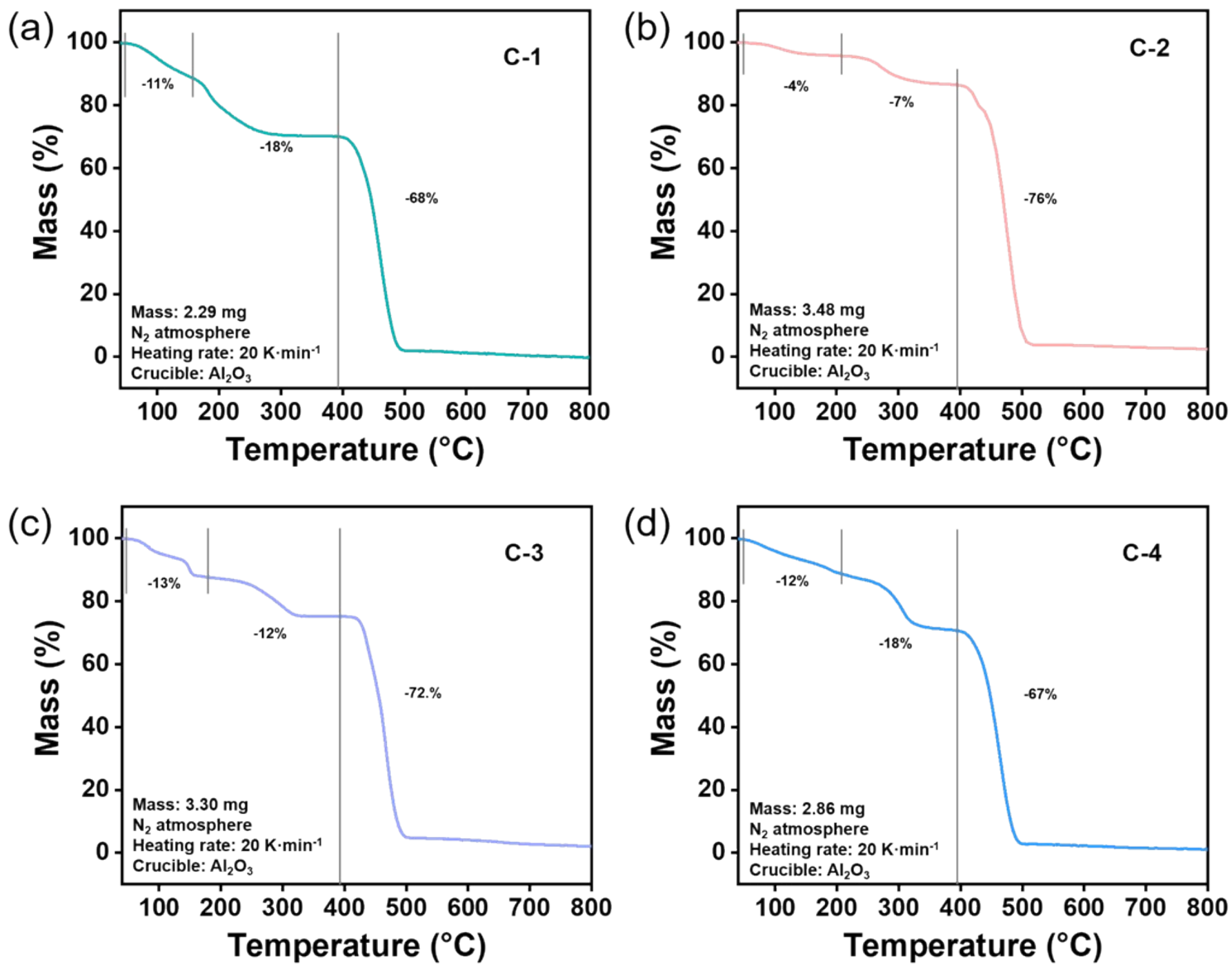

Figure S5. Thermogravimetric Analysis (TGA) curves of (a) C-1, (b) C-2, (c) C-3, and (d) C-4. Taking the *R* enantiomer as an example.

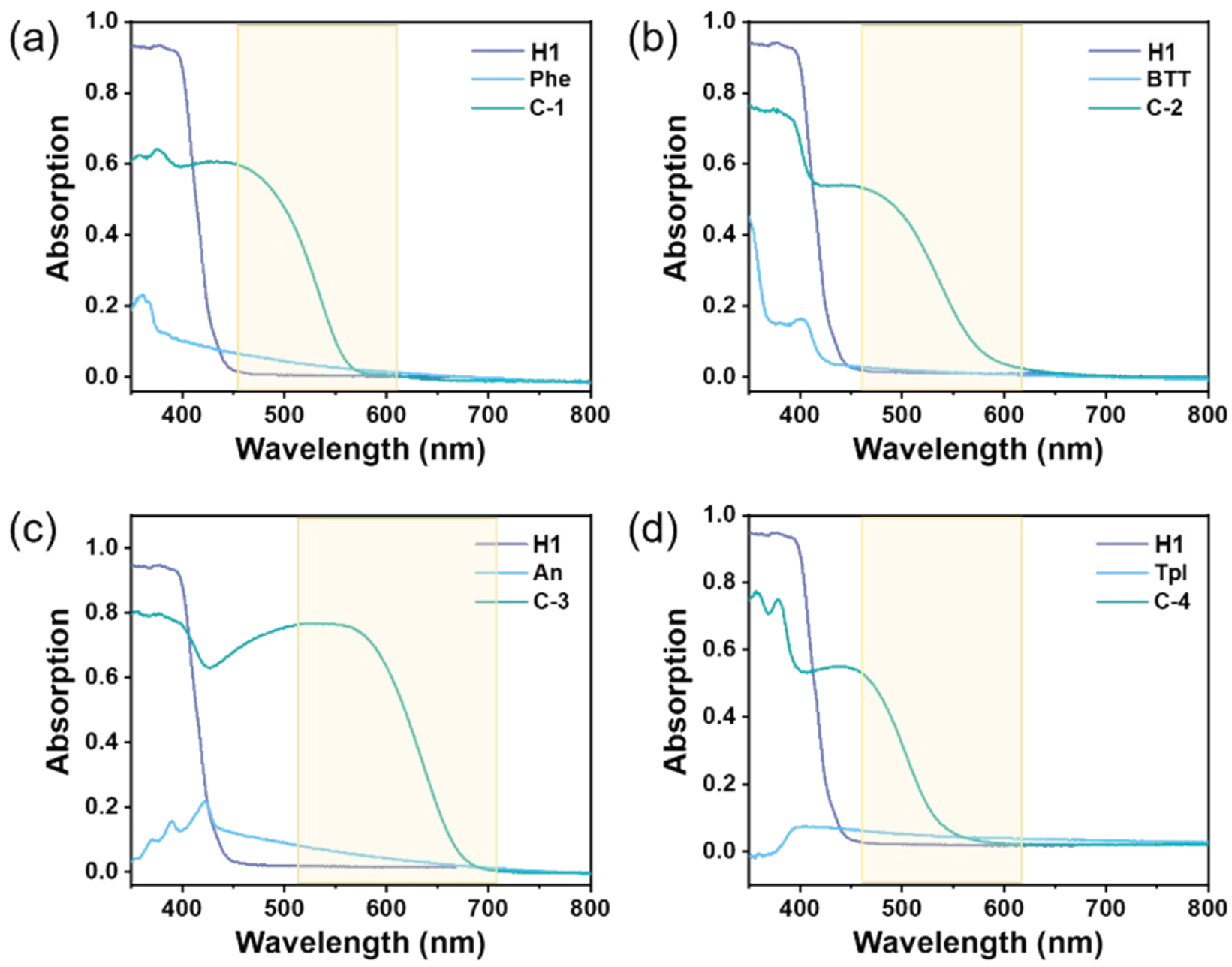

Figure S6. The solid-state ultraviolet-visible (UV-vis) absorption spectra of monomer and co-crystals. (a) H1, Phe, and C-1; (b) H1, BTT, and C-2. (c) H1, An, and C-3; (d) H1, Tpl, and C-4. The UV absorption edge of the red species is highlighted in light yellow. Taking the *R* enantiomer as an example.

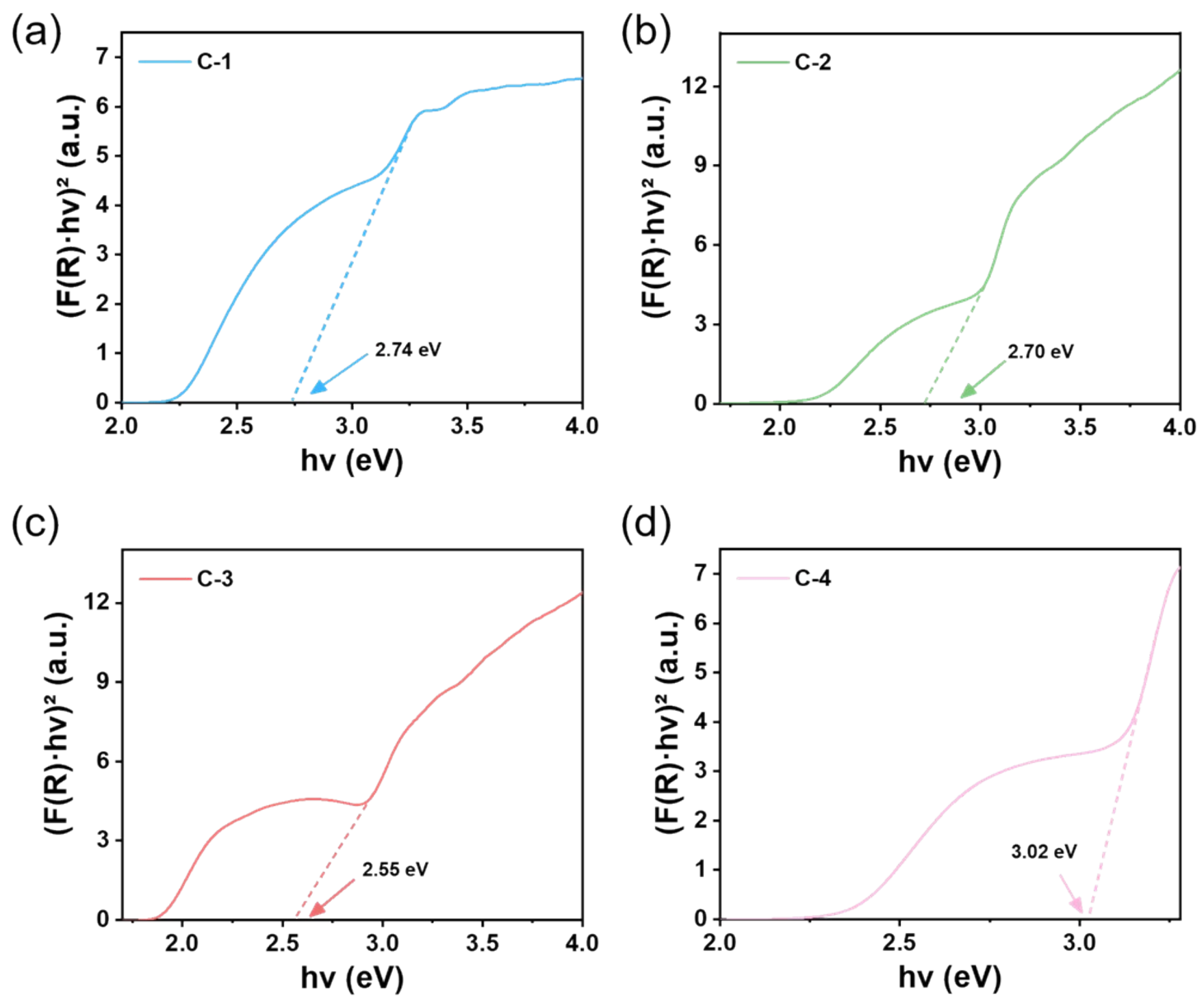

Figure S7. Tauc plots of (F(R)·hν)^2 versus photon energy (hν) derived from solid-state UV–vis diffuse reflectance spectra for (a) C-1, (b) C-2, (c) C-3, and (d) C-4 cocrystals. The optical band gaps were determined by linear extrapolation of the absorption edge. Taking the *R* enantiomer as an example.

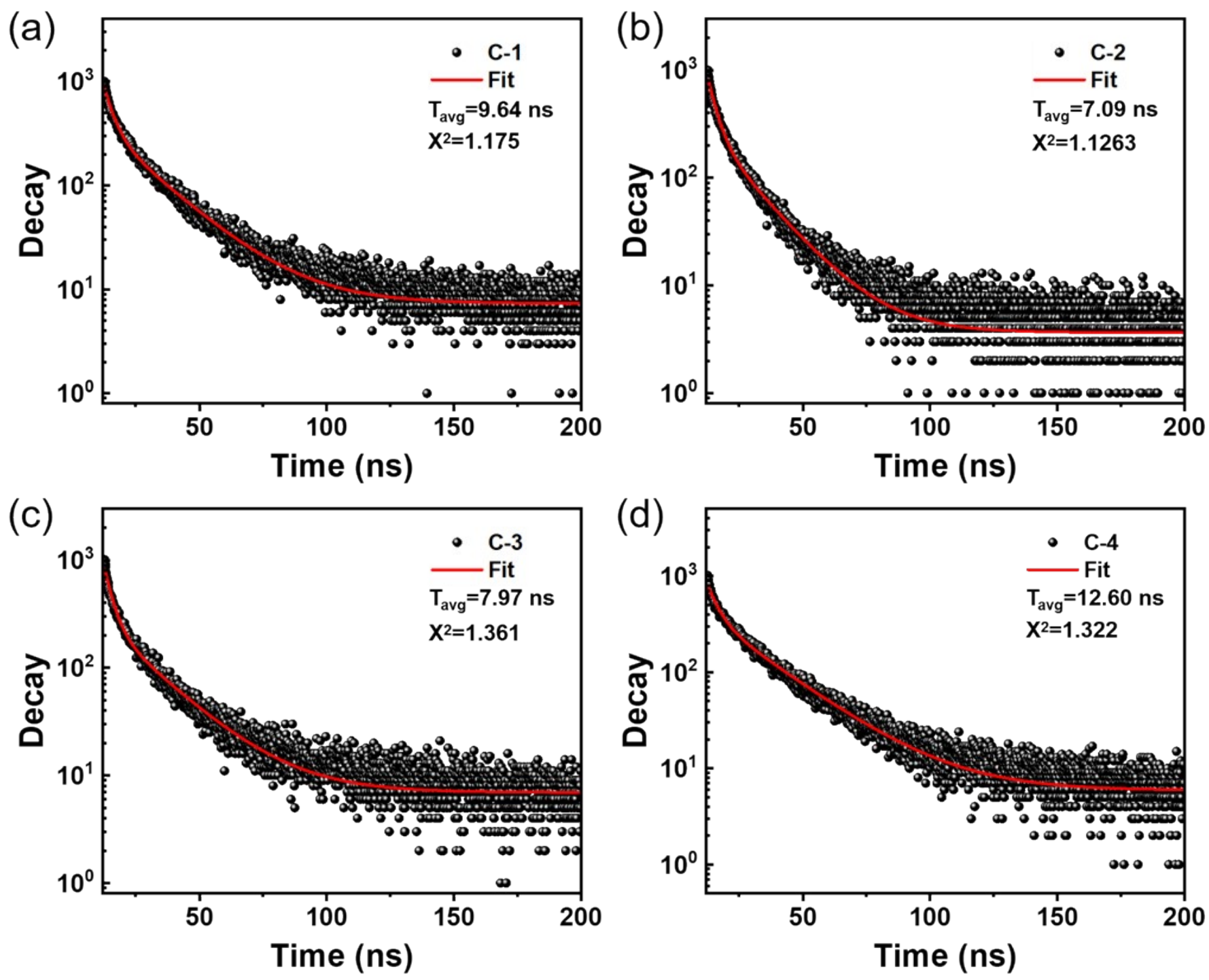

Figure S8. The fluorescence decay curves and lifetime for (a) C-1, (b) C-2, (c) C-3, and (d) C-4. λ_{ex} = 405 nm. Taking the *R* enantiomer as an example.

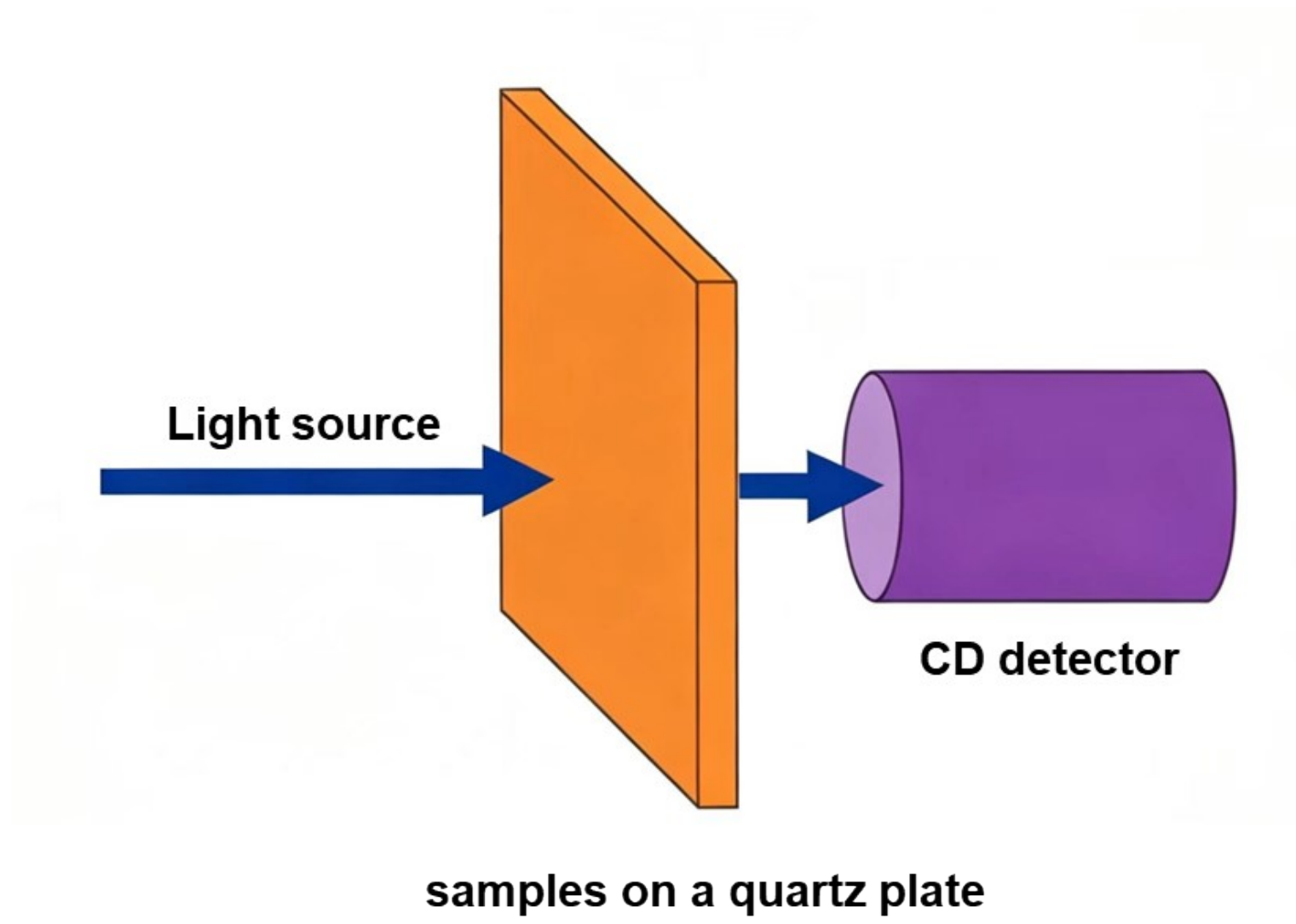

Figure S9. A diagram of the set up for measuring the CD of drop-casting film (side view).

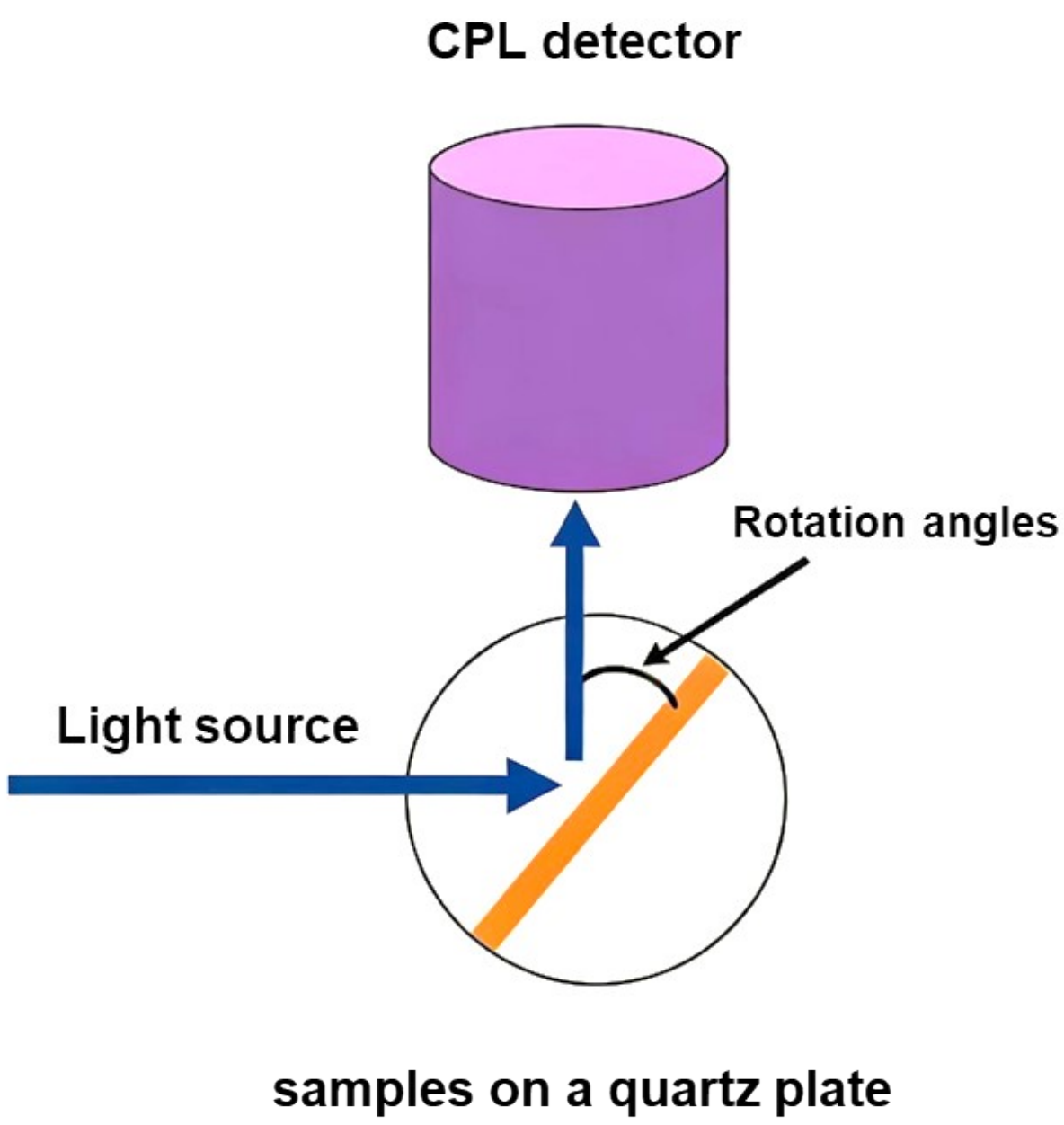

Figure S10. A diagram of the set up for measuring the CPL of drop-casting film (top view).

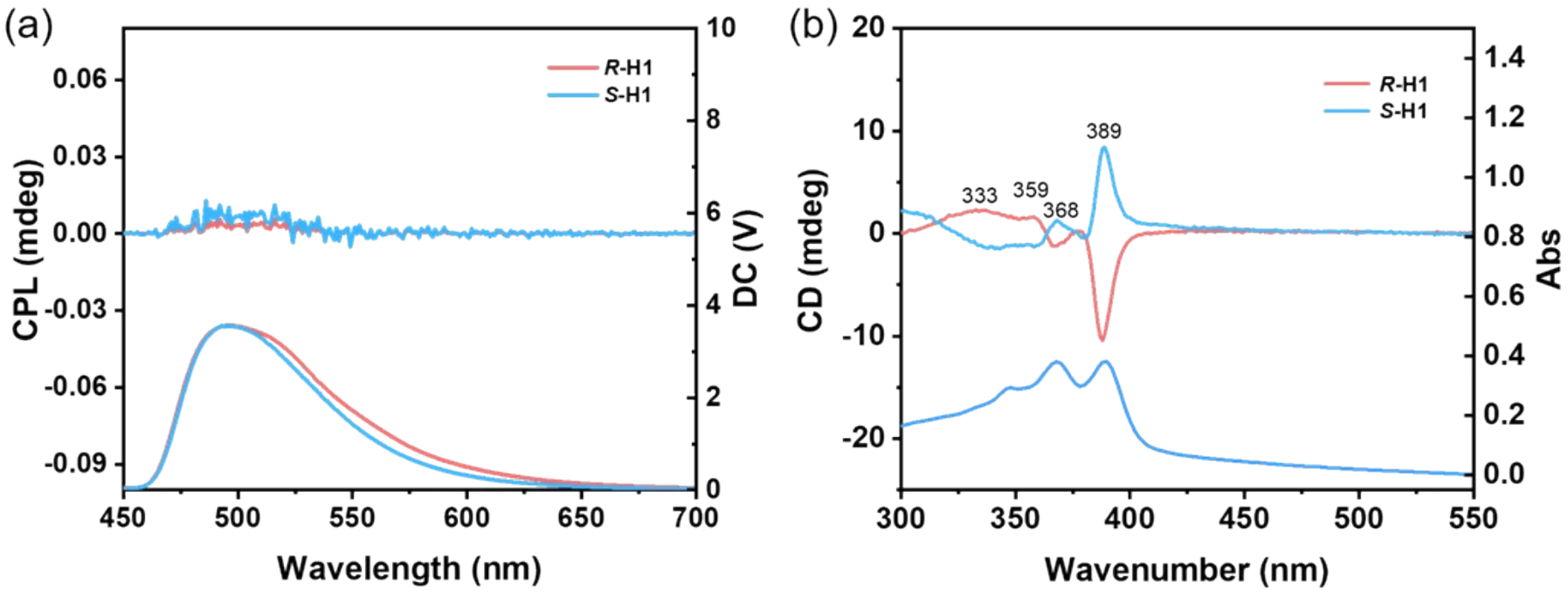

Figure S11. (a) CD and UV spectra of of *R/S*-H1 self-assemblies obtained by drop-casting from CH_2Cl_2. (b) CPL and DC spectra of *R/S*-H1.

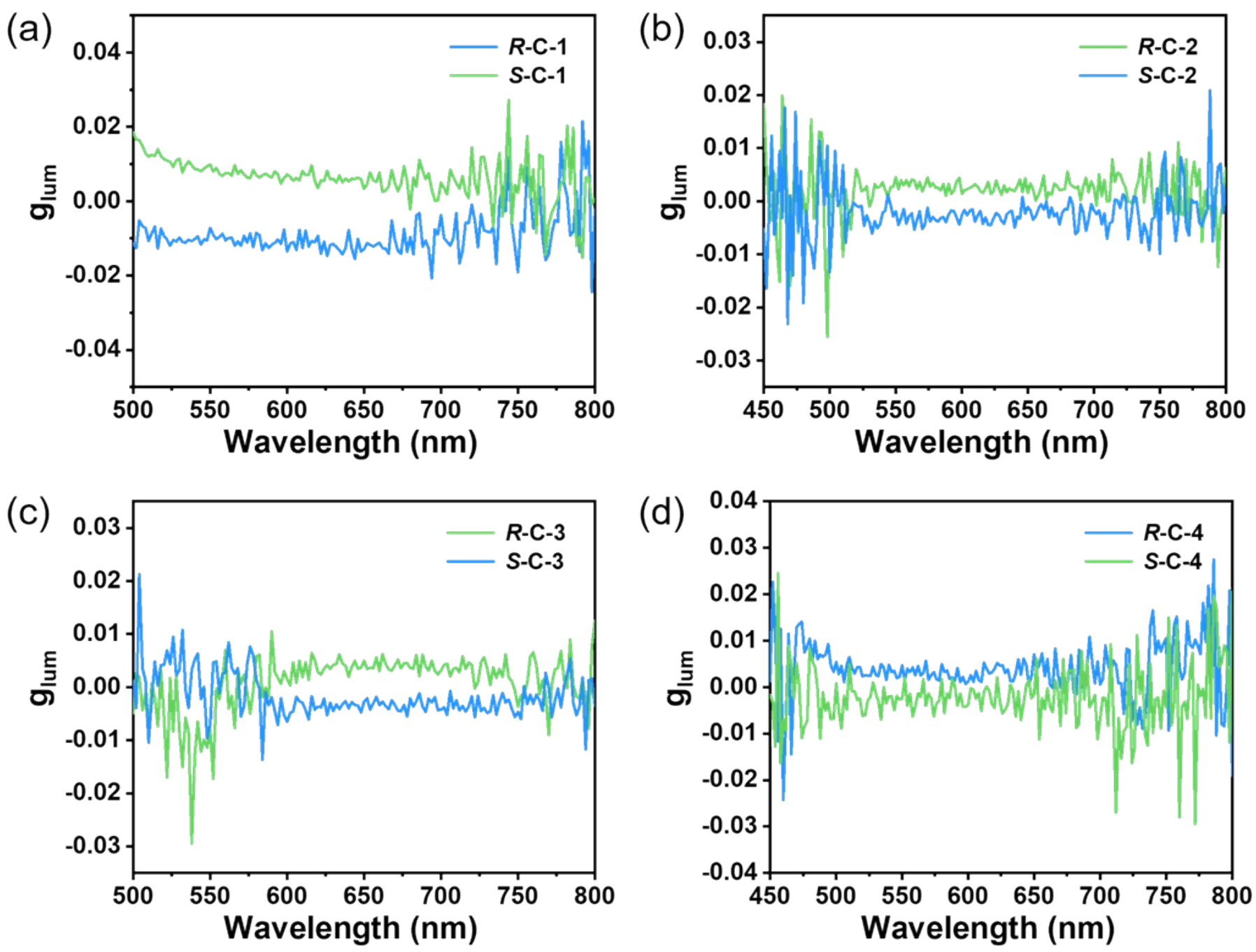

Figure S12. The g_{lum} of (a) *R/S*-C-1, (b)*R/S*- C-2, (c) *R/S*-C-3, and (d) *R/S*-C-4.

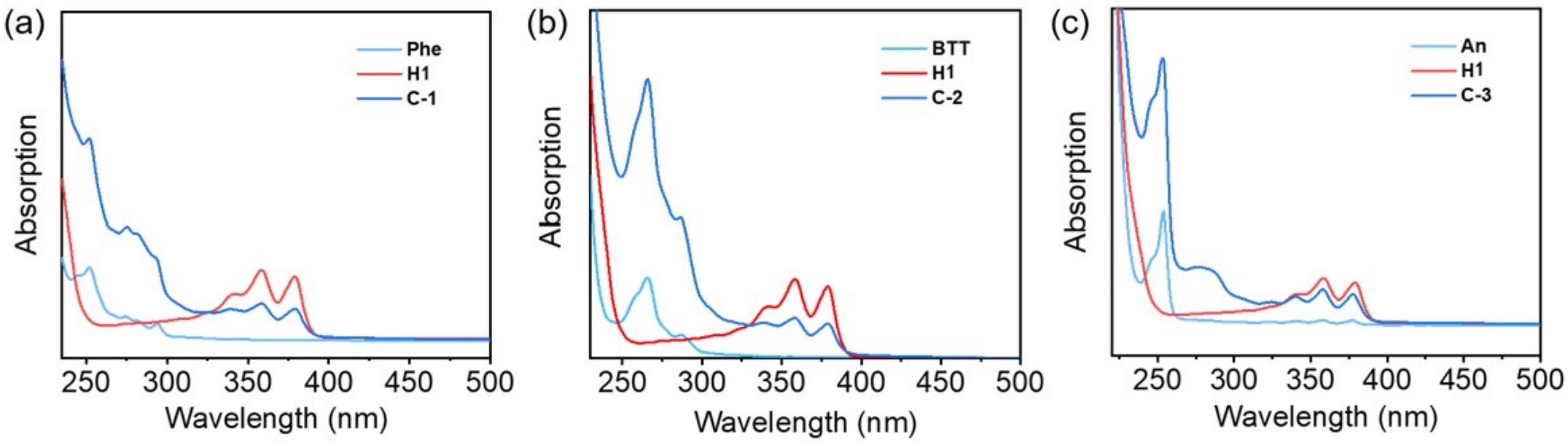

Figure S13 UV–vis absorption spectra of H1 with different guest molecules (An, Phe, and BTT) and their mixture (solution concentration: 1×10^{-5} M). Taking the *R* enantiomer as an example。

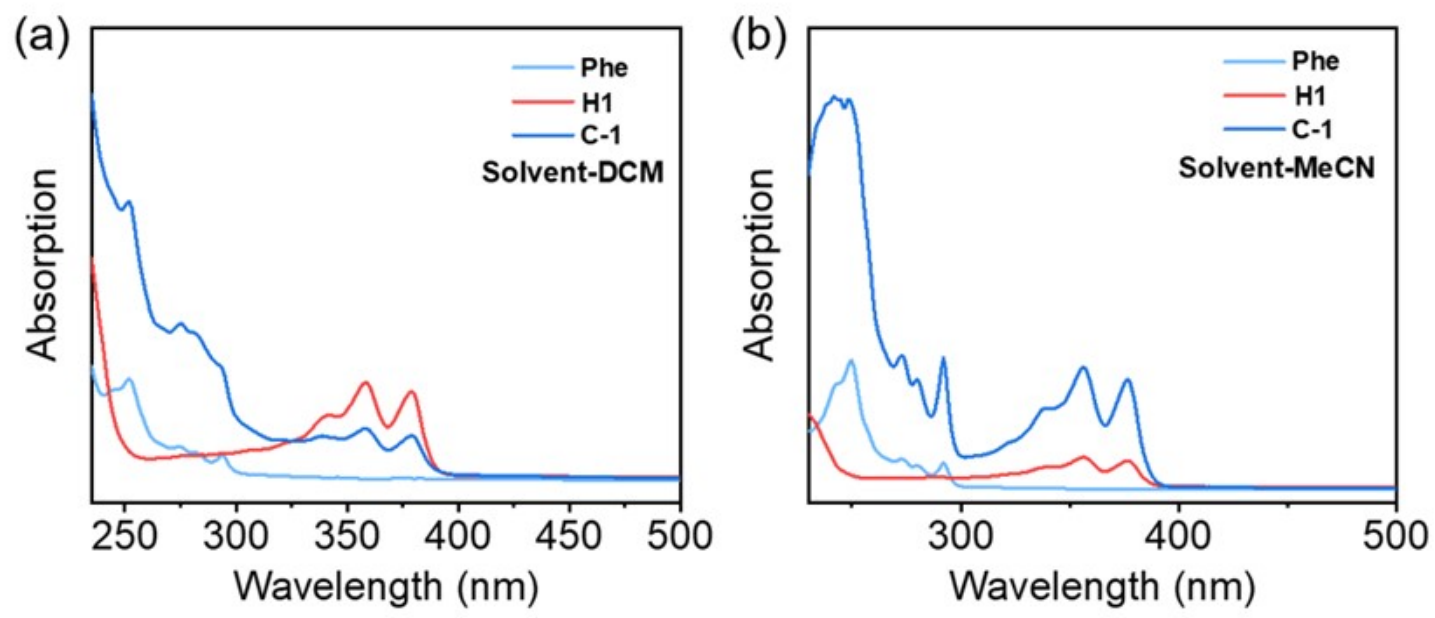

Figure S14 (a) UV–vis absorption spectra of H1, Phe, and their mixture in DCM; (b) UV–vis absorption spectra of H1, Phe, and their mixture in MeCN. The *R* enantiomer is shown as a representation.

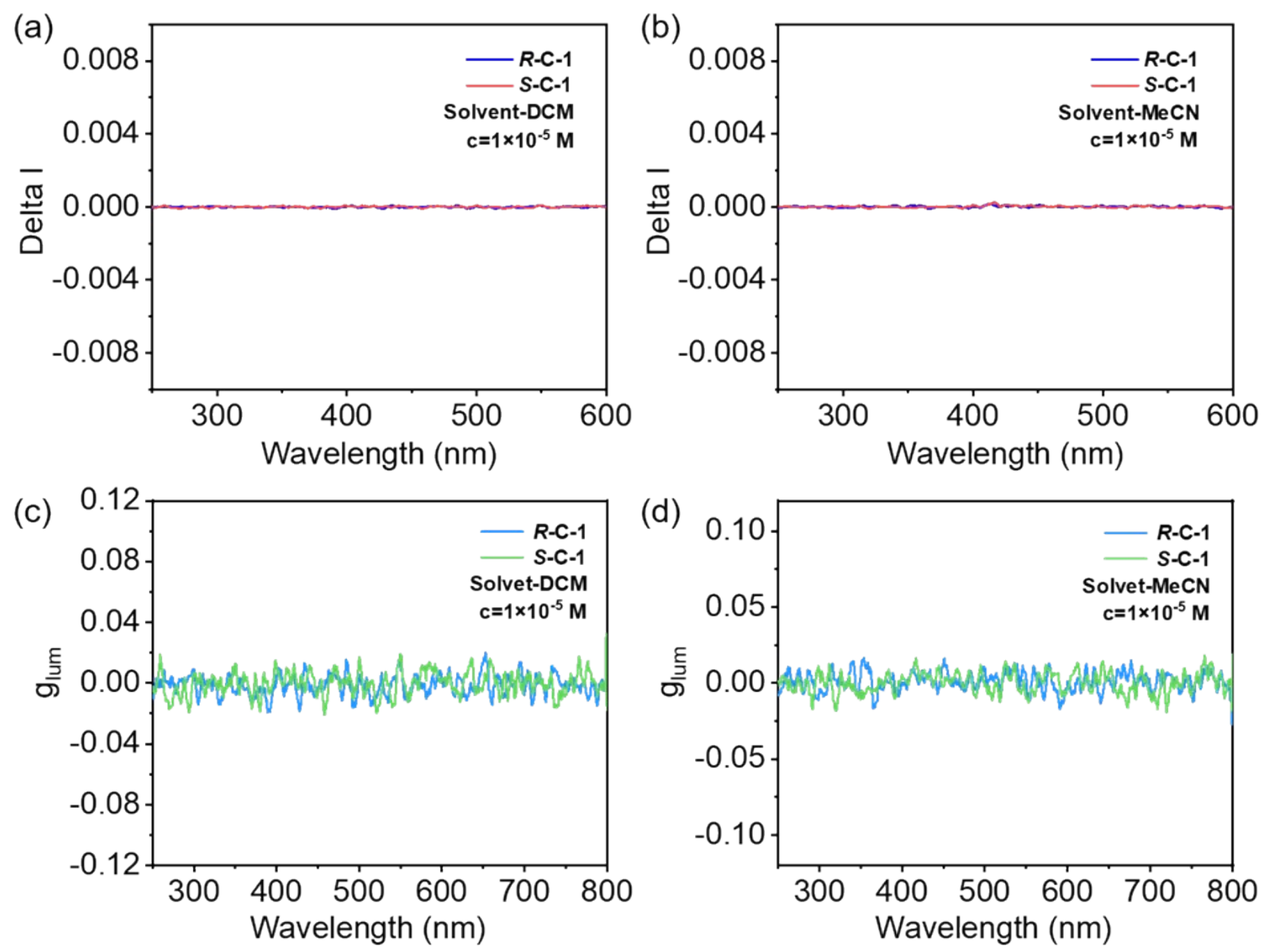

Figure S15 (a) CPL spectra of *R/S*-C-1 (R/S-H1–phenanthrene) in DCM; (b) CPL spectra of *R/S*-C-1 in MeCN; (c) g_{lum} values of *R/S*-C-1 in DCM; (d) g_{lum} values of *R/S*-C-1 in MeCN (solution concentration: 1×10^{-5} M).

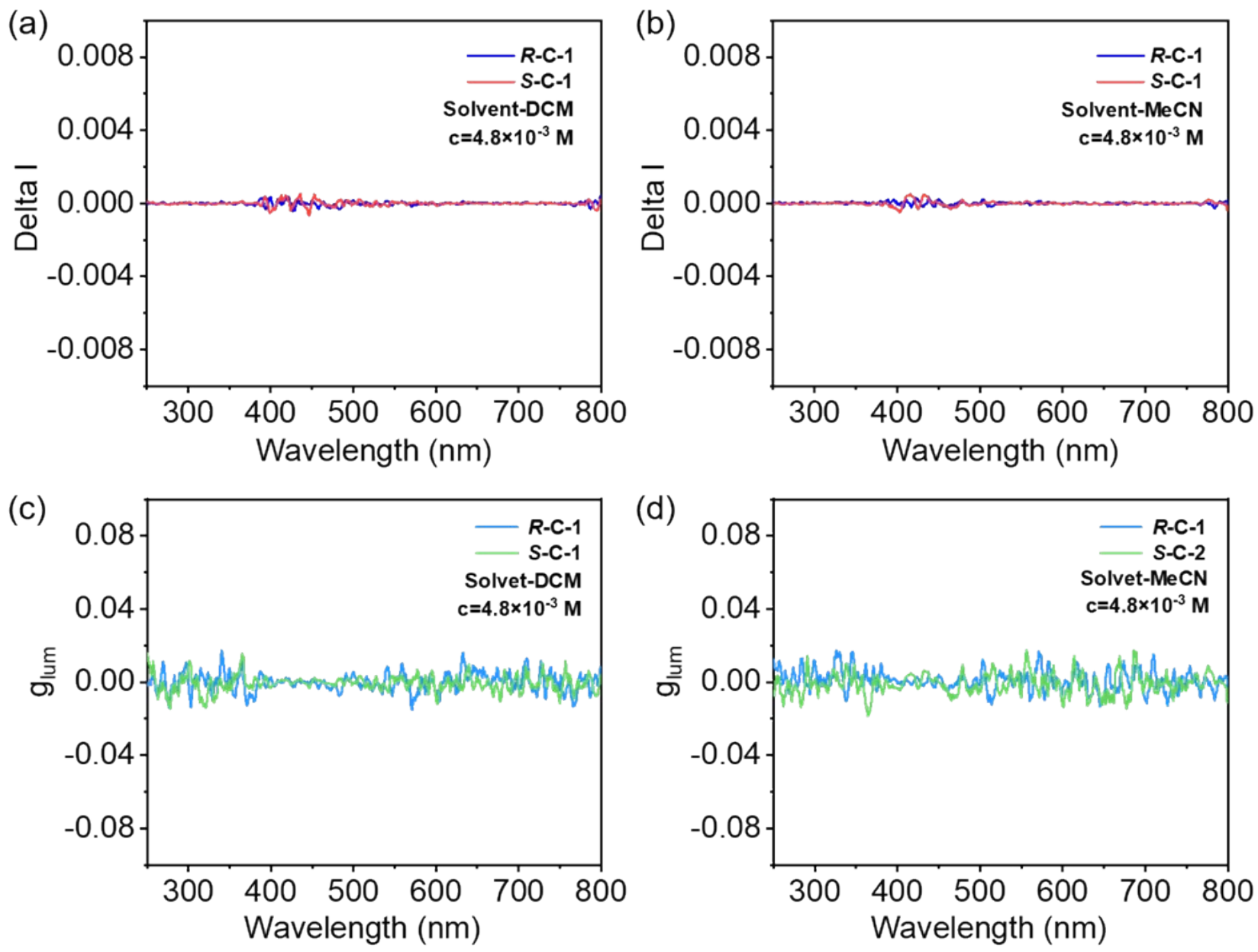

Figure S16 (a) (a) CPL spectra of *R/S*-C-1 in DCM; (b) CPL spectra of *R/S*-C-1 in MeCN; (c) g_{lum} values of *R/S*-C-1 in DCM; (d) g_{lum} values of *R/S*-C-1 in MeCN (solution concentration: 4.8 × 10^{-3} M).

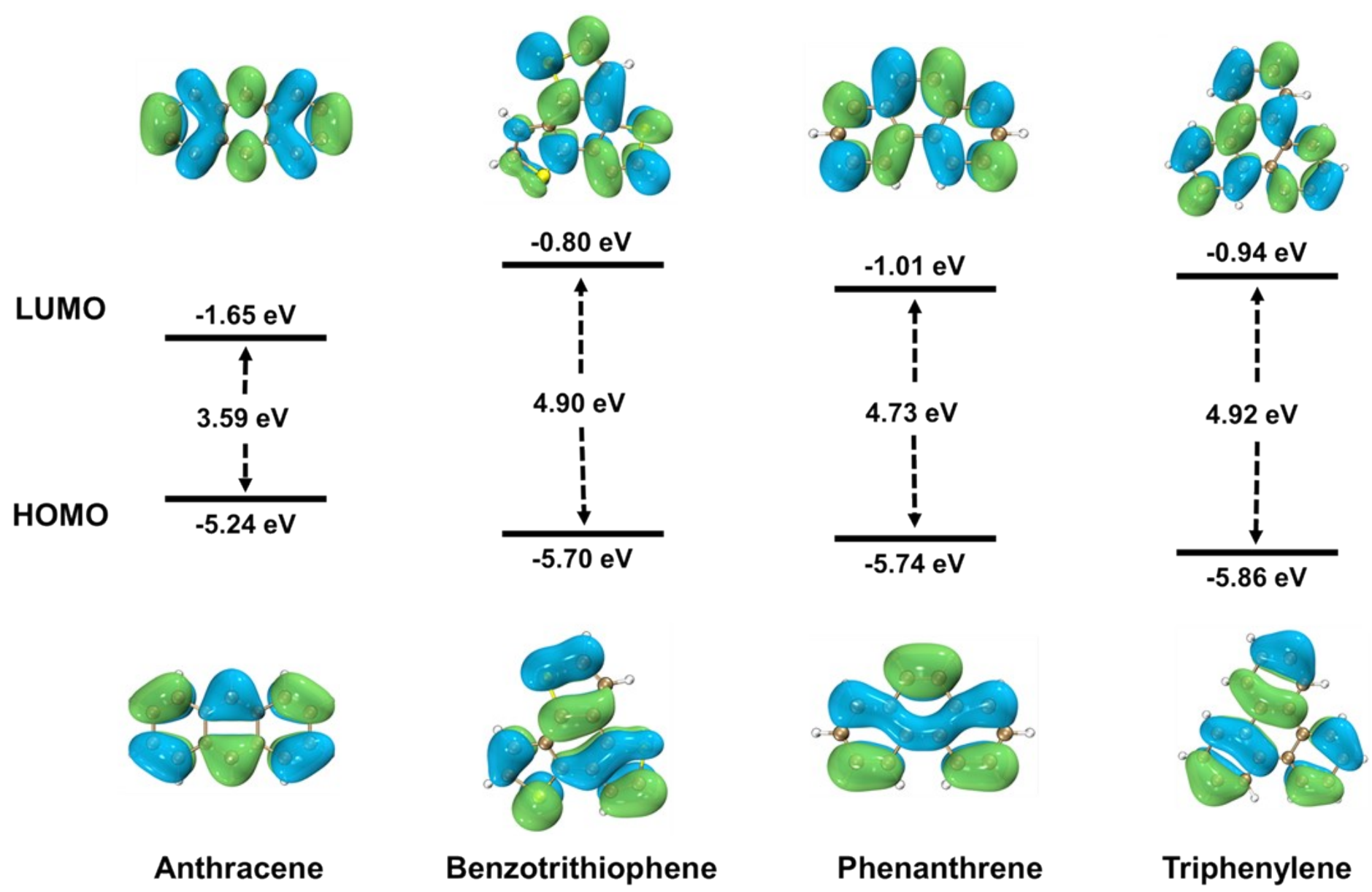

Figure S17. The HOMOs, LUMOs and HOMO-LUMO energy gaps of phenanthrene, benzotrithiophene, triphenylene, and anthracene obtained through DFT calculations.

3. Reference

1. Schneebeli, S. T.; Frasconi, M.; Liu, Z.; Wu, Y.; Gardner, D. M.; Strutt, N. L.; Cheng, C.; Carmieli, R.; Wasielewski, M. R.; Stoddart, J. F., Electron sharing and anion-pi recognition in molecular triangular prisms. *Angew. Chem. Int. Ed.* 2013, **52**, 13100-13104.

2. Frisch, M. J.; Trucks, G. W.; Schlegel, H. B.; Scuseria, G. E.; Robb, M. A.; Cheeseman, J. R.; Scalmani, G.; Barone, V.; Petersson, G. A.; Nakatsuji, H.; Li, X.; Caricato, M.; Marenich, A. V.; Bloino, J.; Janesko, B. G.; Gomperts, R.; Mennucci, B.; Hratchian, H. P.; Ortiz, J. V.; Izmaylov, A. F.; Sonnenberg, J. L.; Williams; Ding, F.; Lipparini, F.; Egidi, F.; Goings, J.; Peng, B.; Petrone, A.; Henderson, T.; Ranasinghe, D.; Zakrzewski, V. G.; Gao, J.; Rega, N.; Zheng, G.; Liang, W.; Hada, M.; Ehara, M.; Toyota, K.; Fukuda, R.; Hasegawa, J.; Ishida, M.; Nakajima, T.; Honda, Y.; Kitao, O.; Nakai, H.; Vreven, T.; Throssell, K.; Montgomery Jr., J. A.; Peralta, J. E.; Ogliaro, F.; Bearpark, M. J.; Heyd, J. J.; Brothers, E. N.; Kudin, K. N.; Staroverov, V. N.; Keith, T. A.; Kobayashi, R.; Normand, J.; Raghavachari, K.; Rendell, A. P.; Burant, J. C.; Iyengar, S. S.; Tomasi, J.; Cossi, M.; Millam, J. M.; Klene, M.; Adamo, C.; Cammi, R.; Ochterski, J. W.; Martin, R. L.; Morokuma, K.; Farkas, O.; Foresman, J. B.; Fox, D. J. *Gaussian 16 Rev. C.01*, Wallingford, CT, 2016.
3. Lu, T.; Chen, F., Multiwfn: A multifunctional wavefunction analyzer. *J. Comput. Chem.* 2012, **33**, 580-592.
